# Efficient hybrid de novo assembly of human genomes with WENGAN

**DOI:** 10.1038/s41587-020-00747-w

**Published:** 2020-12-14

**Authors:** Alex Di Genova, Elena Buena-Atienza, Stephan Ossowski, Marie-France Sagot

**Affiliations:** 1 Inria Grenoble Rhône-Alpes, Montbonnot, France; 2 Université de Lyon, Université Lyon 1, CNRS, Laboratoire de Biométrie et Biologie Evolutive UMR 5558, Villeurbanne, France; 3 Institute of Medical Genetics and Applied Genomics, University of Tübingen, Tübingen, Germany; 4 NGS Competence Center Tübingen (NCCT), University of Tübingen, Tübingen, Germany

**Keywords:** Computational biology and bioinformatics, Software, Genomics, Genome assembly algorithms

## Abstract

Generating accurate genome assemblies of large, repeat-rich human genomes has proved difficult using only long, error-prone reads, and most human genomes assembled from long reads add accurate short reads to polish the consensus sequence. Here we report an algorithm for hybrid assembly, WENGAN, that provides very high quality at low computational cost. We demonstrate de novo assembly of four human genomes using a combination of sequencing data generated on ONT PromethION, PacBio Sequel, Illumina and MGI technology. WENGAN implements efficient algorithms to improve assembly contiguity as well as consensus quality. The resulting genome assemblies have high contiguity (contig NG50: 17.24–80.64 Mb), few assembly errors (contig NGA50: 11.8–59.59 Mb), good consensus quality (QV: 27.84–42.88) and high gene completeness (BUSCO complete: 94.6–95.2%), while consuming low computational resources (CPU hours: 187–1,200). In particular, the WENGAN assembly of the haploid CHM13 sample achieved a contig NG50 of 80.64 Mb (NGA50: 59.59 Mb), which surpasses the contiguity of the current human reference genome (GRCh38 contig NG50: 57.88 Mb).

## Abstract (web summary)

The genome assembler WENGAN produces high-quality human genome sequences at low computational cost.

## Main

Genome assembly is the process by which an unknown genome sequence is constructed by detecting overlaps between a set of redundant genomic reads. Most genome assemblers represent the overlap information using different kinds of assembly graph^1,2^. The main idea behind these algorithms is to reduce the genome assembly problem to a path problem where the genome is reconstructed by finding the true genome path in a tangled assembly graph^1,2^. The entanglement comes from the complexity that repetitive genomic regions induce in the assembly graphs^1,2^. The first graph-based genome assemblers used overlaps of variable length to construct an overlap graph^2^. The main goal of the overlap graph approach and of its subsequent evolution, namely the string graph^3^, is to preserve the read information^2,3^. However, read-level graph construction requires an expensive all-versus-all read comparison^3^. The read-level nature implies that a path in such a graph represents a read layout, and a subsequent consensus step must be performed to improve the quality of bases called along the path^3^. These graph properties are the foundation of the overlap–layout–consensus (OLC) paradigm^3–5^.

A seemingly counterintuitive idea is to fix the overlap length to a given size (*k*) to build a de Bruijn graph^1^. However, de Bruijn graphs have several favorable properties making them the method of choice in many modern short-read assemblers^6–8^. In this approach, the fixed-length exact overlaps are detected by breaking the reads into consecutive *k*-mers^1^. The *k*-mers are usually stored in hash tables (constant query time), thus avoiding entirely the costly all-versus-all read comparison^6–8^. Unlike a string graph, the de Bruijn graph is a base-level graph^1,6–8^; thus, a path (contig) represents a consensus sequence derived from a pileup of the reads generating the *k*-mers (*k*-mer frequency). Moreover, the de Bruijn graph is useful for characterizing repeated as well as unique sequences of a genome (repeat graph^9^). However, by splitting the reads into *k*-mers, valuable information from the reads may be lost, especially when these are much longer than the selected *k*-mer size^3^.

The type of overlap detected, and therefore the type of assembly graph constructed, is related to the sequencing technology used to generate the reads. One class of modern high-throughput sequencing machines produces short (100–300 base pairs (bp)) and accurate (base error < 0.1%) reads^10,11^, and a second class produces long (>10 kilobases (kb)) but error-prone (base error < 15%) reads^12,13^. Despite the high per-base error rate of long reads, these are the better choice for genome reconstruction^14^, as longer overlaps reduce the complexity of the assembly graph^15^, and therefore more contiguous genome reconstructions are achievable^14^.

Regardless of the sequencing technology, the goals of a genome assembler are to reconstruct the complete genome in (1) the fewest possible consecutive pieces (ideally chromosomes) with (2) the highest base accuracy while (3) minimizing the computational resources (the 1–2–3 goals). Short-read de Bruijn graph assemblers are good for accomplishing goals 2 and 3 (refs. ^6–8^), while long-read assemblers excel at achieving goal 1 (refs. ^4,5^).

Modern long-read assemblers widely adopted the OLC paradigm^4,5,16–19^ and new algorithms have substantially accelerated the all-versus-all read comparison^16–19^. Such progress has been possible by avoiding entirely the long-read error-correction step^16–19^, and by representing the long reads as fingerprints derived from a subset of special *k*-mers (that is, minimizers^20^, minhash^19^ and so on). The reduced long-read representation is appropriate for detecting overlaps >2 kb in a fast way^16,18,19^. The newest long-read assemblers are therefore starting to be good also at goal 3 (refs. ^16,18,19^). However, assembling uncorrected long reads has the undesirable effect of giving more work to the consensus polisher^17,19,21–23^. Genome assembly polishing is the process of improving the base accuracy of the assembled contig sequences^17,19,21–24^. Usually, long-read assemblers perform a single round of long-read polishing^16,18,19^, which is followed by several rounds of polishing with long^17,19,21,23^ and short^17,22,24^ reads using third-party tools^17,19,21–24^.

Currently, polishing large genomes, such as the human genome, can take much more computational time than the long-read assembly itself^16,18,19^. Even after several rounds of polishing, a substantial fraction of consensus errors remains, hampering the subsequent genome analyses such as gene and protein prediction^25^. Lastly, PacBio recently introduced high-fidelity reads (HiFi reads), substantially increasing the base accuracy of long reads^26^. This technology moves the polishing bottleneck up front by generating multiple error-prone reads (10 passes) of circularized fragments (10–20 kb in size)^26^. Each fragment is then computationally corrected to generate a single consensus long read (>10 kb) with high base accuracy (base error < 1%). To fully exploit HiFi reads, new assemblers have been developed^27,28^ that do not require a final polishing phase^28^.

When this assembly approach employs short-read polishing^17,22,24^, then it corresponds to a long-read-first hybrid assembly strategy^29,30^. Another hybrid assembly strategy consists of starting the assembly process with short reads^31^. However, none of the described hybrid strategies employs the short reads to tackle the problem of assembly contiguity; that is, they do not aim at joining two long reads by a short-read contig, and therefore exploit only partially the short-read sequence information.

In this Article we introduce WENGAN, a hybrid genome assembler that, unlike most long-read assemblers, entirely avoids the all-versus-all read comparison, does not follow the OLC paradigm and integrates short reads in the early phases of the assembly process (short-read-first). We validated WENGAN with standard assembly benchmarks. Our results demonstrate that WENGAN optimizes the 1–2–3 goals and is particularly effective at low long-read coverage (15×). Furthermore, we show that the WENGAN assemblies performed by combining ultralong Nanopore reads with short or HiFi reads surpass the contiguity of the current human reference genome.

## Results

### The WENGAN algorithm

**Fig. 1:**
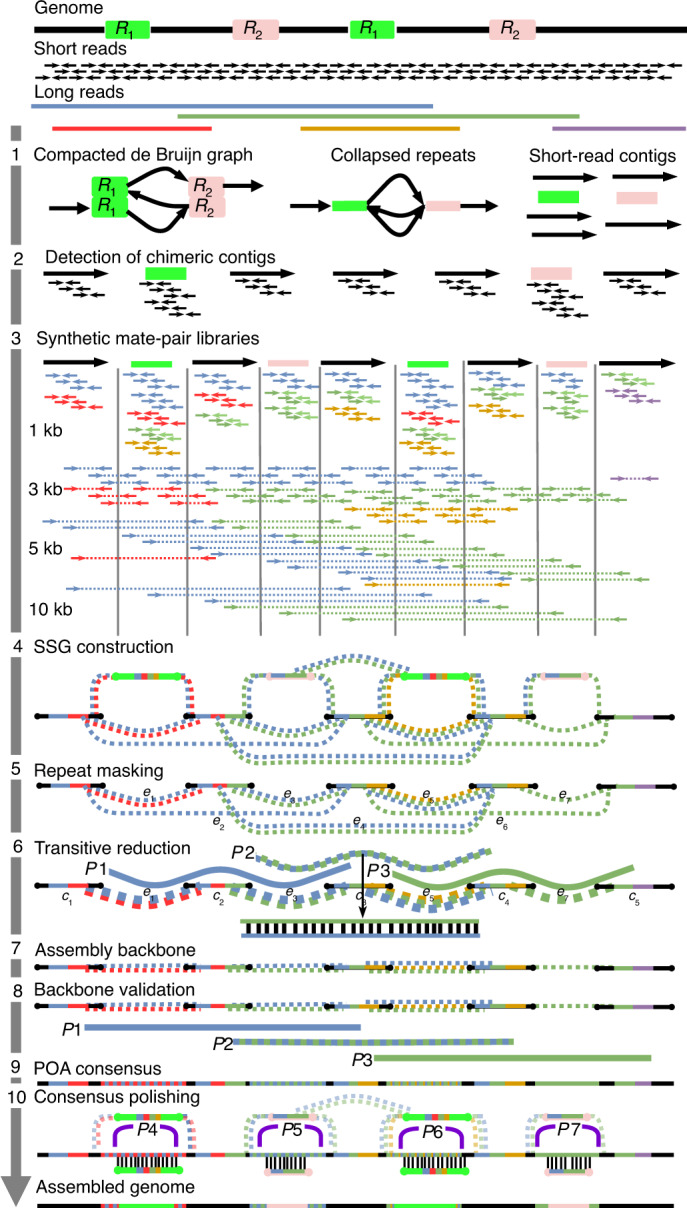
The WENGAN algorithm. The WENGAN workflow consists of first assembling and error-correcting the short-read contigs (1 and 2), creating a spectrum of synthetic mate-pair libraries from long reads (3) and building of the SSG (4). The SSG is used to compute approximate long-read overlaps by building long-read-coherent paths (5 and 6). The long-read overlaps restore the long-read information and facilitate the construction and validation of the assembly backbone (7 and 8). The SSG is used to fill the gaps by building for each mate edge a consensus sequence using the partial order alignment graph (9). In the final step, the SSG is used to polish the consensus sequences (10). The repeat contigs (2–10) are drawn uncollapsed to explain the WENGAN steps.

WENGAN starts by building short-read contigs using a de Bruijn graph assembler^6–8^ (1 in Fig. 1). Then, the pair-end reads are pseudo-aligned^32^ back to detect and error-correct chimeric contigs as well as to classify them as repeats or unique sequences (2 in Fig. 1). Repeated sequences induce complex de Bruijn graph topologies in their neighborhood, and short-read assemblers can choose wrong paths while traversing such complex regions, thus leading to chimeric contigs (Supplementary Fig. 1). Chimeric short-read contigs limit the accuracy and contiguity of the assembly when left uncorrected (Supplementary Fig. 2). Each short-read contig is therefore scanned base-by-base and split at sub-regions lacking pair-end read support (Supplementary Fig. 1).

Following short-read contig correction, we generate synthetic paired reads of different insert sizes from long-read sequences, which are mapped to the corrected short-read contigs (3 in Fig. 1). The spectrum of synthetic libraries is used to span the genomic repeats. For instance, with ultralong Nanopore reads, we can create a spectrum composed of 24 synthetic libraries with insert sizes ranging from 0.5 kb to 200 kb (Supplementary Fig. 3). Matched pairs are stored with a reference to the long read from which they were extracted (colors appearing in pairs; 3 in Fig. 1). Using the mapped pairs and the corrected short-read contigs, we then build the synthetic scaffolding graph (SSG). The SSG is an extension of the scaffolding graph^33^, where there is an additional edge-labeling function that labels (colors) the SSG edges with the long reads (3 and 4 in Fig. 1). After the SSG construction (4 in Fig. 1) and subsequent repeat masking (5 in Fig. 1), we employ the SSG to compute implicit approximate long-read multiple alignments by searching for transitive long-read-coherent paths (6 in Fig. 1). The aim of this graph operation (called transitive reduction) is to restore the full long-read information in the SSG. Each successful reduction modifies the weight as well as the shape of the SSG (6 in Fig. 1). After restoring the long-read information, we order and orient the short-read contigs by applying an approximation algorithm^34^ that uses all of the connectivity information at once to produce an optimal assembly backbone (7 in Fig. 1). The solution is validated by checking the distance constraints that the reduced long-read-coherent paths impose on the assembly backbone (8 in Fig. 1).

A property of the SSG is that all edges connecting two short-read contigs (called mate edges) are spanned by at least one long read. We therefore use the inner long-read sequence of the synthetic mate pairs that span the mate edge to build a long-read consensus sequence using a partial order alignment graph^17,35^ (9 in Fig. 1). The corresponding short-read contig ends are then aligned^36^ to the mate-edge consensus sequence to determine the correct boundaries, thus filling the gap between the two short-read contigs. We computed the Pearson correlation of the mate-edge length before and after filling the gap for a total of 283,727 mate edges. The correlation is very high (*R*^2^ > 0.99) even for large gaps (>100 kb; Supplementary Fig. 4).

The final steps use the SSG to polish the mate-edge consensus sequences by finding long-read-coherent paths that traverse the repeated regions (that is, *P*4 and *P*6 for *R*_1_; 10 in Fig. 1) or pairwise alignments^36^ between the repetitive short-read contigs and the mate-edge consensus sequences (10 in Fig. 1). Finally, the hybrid contigs are reported in FASTA format (Fig. 1).

### WENGAN surpasses the contiguity of GRCh38

To explore the contiguity limit of WENGAN, we assembled the human haploid cell line CHM13, which has been sequenced with a plethora of technologies including accurate short Illumina reads, long and accurate PacBio/HiFi reads^28^ and ultralong Nanopore reads^30^. In particular, the HiFi reads were generated using a large-insert-size (20 kb) library at 30× genome coverage, with half of the HiFi data (N50) contained in accurate reads larger than 17 kb (Supplementary Table 1). Similarly, the Nanopore reads were generated using an ultralong-read protocol optimized for MinION^29^ resulting in 30× genome coverage by reads of at least 100 kb (Supplementary Table 1).

**Fig. 2:**
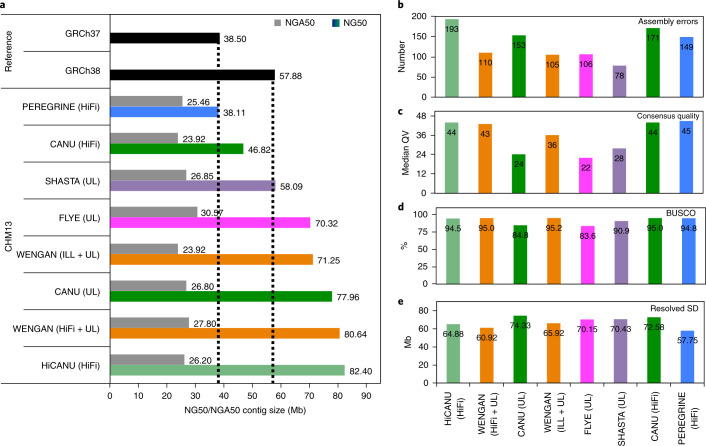
WENGAN assemblies of the haploid CHM13 genome. **a**, A bar plot showing the contig NG50/NGA50 of WENGAN and other state-of-the-art long-read assemblers, as well as of the current human reference genomes. NG50 is the contig length such that using longer contigs produces half (50%) of the bases of the reference genome. NGA50 is NG50 corrected of assembly errors. NG50 and NGA50 were computed using as genome size the total contig lengths of GRCh38 (2.94 Gb). **b**, Assembly errors predicted by QUAST using the GRCh38 reference (autosomes plus X and Y). Assembly errors overlapping centromeric regions or SDs were excluded from the analysis. **c**, Consensus quality assessment by alignment of 30 unique BAC sequences to the assembled contigs using the BACVALIDATION tool. **d**, Gene completeness was determined using the BUSCO tool. **e**, SDs resolved by the genome assemblies. An SD is considered resolved if the aligned contig extends the SD flanking sequences by at least 50 kb (see Methods). Different CHM13 assemblers are represented using the same color across **a**–**e**. Source data

We generated two WENGAN assemblies, one that combines 60× Illumina reads (2 × 250 bp; Supplementary Table 2) with ultralong Nanopore reads, termed WENGAN (ILL + UL), and a second one that combines both long-read technologies, termed WENGAN (HiFi + UL). The WENGAN (ILL + UL) assembly has a total length of 2.84 Gb with half of the genome contained in contig sequences larger than 71.25 Mb (NG50; Fig. 2a). Similarly, the WENGAN (HiFi + UL) assembly has a total length of 2.84 Gb with a contig NG50 of 80.64 Mb (Fig. 2a). The contig NG50 values of both WENGAN assemblies exceed the contiguity of the human reference genomes GRCh37 and GRCh38 (Fig. 2a and Supplementary Fig. 5).

We compared WENGAN to state-of-the-art non-hybrid long-read assemblers (Fig. 2) using public assemblies generated from ultralong Nanopore^5,18,19,30^ or PacBio/HiFi reads^5,27,28^ (see Supplementary Table 3 and the Assembly validation section in the Methods). These genome assemblies of CHM13 represent the quality that can be achieved using the two long-read technologies independently. In terms of assembly contiguity, the NG50 of WENGAN (ILL + UL) is almost twice as long compared to PEREGRINE (HiFi)^27^ (NG50: 38.11 Mb) and CANU (HiFi)^5^ (NG50: 46.82 Mb), is substantially longer than the assembly generated by SHASTA (UL)^19^ (NG50: 58.09 Mb) and has a similar NG50 to the assemblies generated by FLYE (UL)^18^ (NG50: 70.32 Mb) and CANU (UL)^5^ (NG50: 77.96 Mb). The WENGAN (HiFi + UL) assembly reaches an NG50 of 80.64 Mb, which outperforms all aforementioned assemblers, except for the recently developed HiCANU (HiFi) assembler^28^ (NG50: 82.40 Mb; Fig. 2a). An assessment of the assembly quality with QUAST^37^ based on a whole-genome alignment to the GRCh38 reference and subsequent masking of complex genomic regions (see Methods) reveals that both WENGAN assemblies have a low rate of assembly errors (average: 107.5; Fig. 2b), which is comparable or lower than its peers, except for SHASTA (78 errors). Replacing the GRCh38 reference by the curated CHM13 assembly generated by the T2T consortium (v.0.7)^30^ confirms the low error rate achieved by WENGAN (Supplementary Table 4).

We evaluated the consensus quality of the assemblies using an independent set of bacterial artificial chromosome (BAC) sequences of CHM13 located in unique genomic regions^30^ (Supplementary Table 5). Our analysis shows that WENGAN (ILL + UL) and WENGAN (HiFi + UL) assemblies achieved median consensus qualities (median QV ≥ 36.06 and QV ≥ 42.88) that exceed the base quality of Nanopore assemblers, and are comparable to the base qualities of HiFi assemblers (Fig. 2c). Moreover, the WENGAN and HiFi assemblies excel at BUSCO completeness with a recovery of at least 94.5% of the BUSCO genes (Fig. 2d). In terms of computational resources, WENGAN (ILL + UL) took 1,198 CPU hours (maximum RAM 646 Gb, 38 h real time). The run time of WENGAN was at least 183 times faster than that of CANU (UL) (~219,000 CPU hours)^19^, while at the same time using less memory than other assemblers such as FLYE and SHASTA. Interestingly, generating HiFi consensus reads for 30× human genome coverage requires ~40,000 CPU hours (ref. ^28^), which is ~40 times more computationally intensive than the WENGAN (ILL + UL) de novo assembly. Disregarding the excessive generation time for HiFi reads, WENGAN (HiFi + UL) took 981 CPU hours (maximum RAM 125 Gb, 85 h real time), which is more efficient than HiCANU (5,000 CPU hours)^28^, but less efficient than PEREGRINE (58 CPU hours)^28^.

We assessed the performance of the assemblers in hard-to-assemble regions such as the repeat sequences annotated in the curated CHM13 T2T-X chromosome^30^, the major histocompatibility complex (MHC) and segmental duplications (SDs). The T2T-X chromosome (154 Mb, v.0.7) is the first human chromosome completely assembled^30^, and thus is useful to assess the performance of assemblers across all of the repeat families. The MHC region is repetitive and highly polymorphic^29^, while SDs are the most complex repeats annotated in the human genome^38^ with more than 100 Mb of the SD sequence composed of repeats larger than 100 kb (Supplementary Fig. 6a). The T2T-X chromosome is covered by 2 and 4 contigs with a total size of 150.9 Mb and 150.56 Mb in WENGAN (HiFi + UL) and WENGAN (ILL + UL), respectively (Supplementary Fig. 7). Both WENGAN assemblies solve more than 99.6% of the total interspersed repeats annotated in the curated T2T-X chromosome, which is better than or comparable to its peers (Supplementary Table 6). All evaluated CHM13 assemblies span the 4.97 Mb MHC region in a single contig (Supplementary Fig. 8), with the WENGAN assemblies reaching an NGA50 of 2.8 Mb (Supplementary Fig. 8). The WENGAN assemblies resolve between 168 and 176 BAC sequences (Supplementary Table 5), which is better than PEREGRINE (136), comparable to SHASTA (176) and lower than FLYE (253), CANU (314) and HiCANU (326). While the BAC library is enriched in SDs^30^, it does not represent the full range of SDs annotated in GRCh38 (175 Mb). The WENGAN assemblies resolve between 60.9 and 65.9 Mb (Fig. 2e) of the SDs annotated in GRCh38 (ref. ^38^), which is better than PEREGRINE, comparable to HiCANU and lower than FLYE, SHASTA and CANU (Supplementary Fig. 6). However, none of the assemblers resolved more than 42% of such hard-to-assemble regions, with the best performer assembling just 22% (CANU (UL): 23.4 Mb) of the SDs ≥100 kb (104.7 Mb; Supplementary Fig. 6). Even with ultralong reads or accurate HiFi reads, a further improvement of the algorithmic approaches will be necessary to complete the assembly of SDs^38^.

Overall, we demonstrated that WENGAN achieved a genome assembly quality that rivals the curated CHM13 assembly (v.0.7) generated by the T2T consortium^30^. Furthermore, replacing the PacBio/HiFi reads for short reads produced a highly competitive assembly contiguity and quality.

### Evaluation of assembly accuracy and contiguity using BIONANO optical mapping

**Fig. 3:**
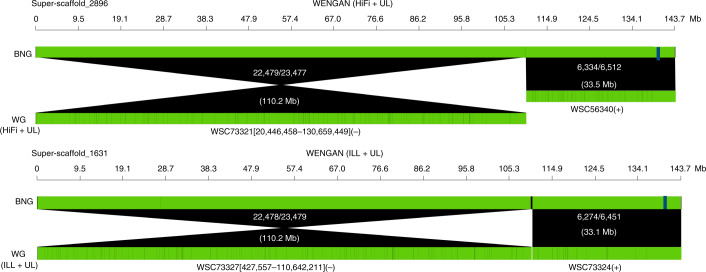
BIONANO scaffolding of the WENGAN assemblies of CHM13. We show the largest super-scaffold produced by merging the BIONANO map (BNG) and the WENGAN (WG) contigs generated by combining ultralong Nanopore reads (rel3) with PacBio/HiFi (20 kb) or Illumina (2 × 250 bp) reads. The name of the scaffolded WENGAN (WSC) contigs is displayed. The square brackets in the contig name indicate that the contig was corrected by the BIONANO map, and the numbers are the start–stop coordinates of the error-free contig region. In round brackets, we show the contig orientation in the super-scaffold. The white text in the alignments displays the number of matching nicking sites, the total number of nicking sites in the BNG contig and the length in megabases of the alignment. The blue bar in the BNG contigs shows examples of joins guided by the WENGAN contigs. Source data

We observed that the distance between the NG50 and NGA50 values increases at greater assembly contiguity ($\bar x = 39.6{\mathrm{Mb}}$; Fig. 2a), which is likely caused by real sequence variation between the sequenced CHM13 sample and the GRCh38 reference genome. Given this limitation of the reference-based validation, we additionally used an independent de novo BIONANO genome map of CHM13 (ref. ^30^) to assess the correctness of the WENGAN assemblies. The BIONANO map is 2.97 Gb in size with 255 contigs and an N50 of 59.6 Mbp. The BIONANO map is integrated with the sequence assembly by identifying in silico the nicking endonuclease-specific sites on the contig sequences (in silico map) followed by alignment of both maps (Fig. 3). Conflicts between the two maps are identified and resolved, and hybrid scaffolds are generated by using the BIONANO maps to join the contig sequences and vice versa (Fig. 3). A total of 72 cuts at conflicting sites were made in 32 contig sequences of the WENGAN (ILL + UL) assembly, leading to a corrected contig NGA50 of 50.73 Mb. The WENGAN (HiFi + UL) assembly after BIONANO conflict correction has an NGA50 of 59.59 Mb (52 cuts in 24 contigs). Both corrected WENGAN assemblies are more contiguous than the GRCh37 reference genome (Fig. 2a). Notably, the contiguity of the corrected WENGAN (HiFi + UL) assembly surpasses the one of the GRCh38 reference genome (59.59 versus 57.88 Mb; Fig. 2a). The hybrid scaffolding produced a maximum of 102 super-scaffold sequences with a total size of 2.83 Gb and an N50 of at least 80 Mb (Fig. 3) for both WENGAN assemblies. Only 0.8% (maximum: 22.42 Mb) of the WENGAN sequence was not integrated into the hybrid scaffolds (short contigs). The BIONANO scaffolding of CHM13 demonstrates that both unpolished WENGAN assemblies are functional and appropriate for subsequent genome analyses.

### WENGAN optimizes the 1–2–3 de novo assembly goals

To validate WENGAN on diploid human genomes, we assembled three human samples, HG00733, NA24385 and NA12878, which were sequenced with very long reads (Supplementary Table 1). All sequencing data were obtained from public repositories (Supplementary Tables 1 and 2). HG00733 was sequenced using the PacBio Sequel I to 90× genome coverage with N50 ≥ 33.2 kb. NA24385 and NA12878 were sequenced using the Oxford Nanopore Technology (ONT) at 60× and 35× genome coverage and N50s of 54 kb and 72 kb, respectively. The sequence data of NA24385 and NA12878 were generated using an ultralong-read protocol^29^ for ONT MinION and contain at least 3.3× genome coverage in reads larger than 100 kb (Supplementary Table 1). The long-read data were combined with at least 50× short-read coverage (pair ends: 2 × 150 bp or 2 × 250 bp; Supplementary Table 2).

WENGAN was benchmarked in its three assembly modes, namely WENGAN-M (MINIA3)^6^, WENGAN-A (ABYSS2)^7^ and WENGAN-D (DISCOVARdenovo)^8^. We compared WENGAN to six state-of-the-art assemblers (Table 1). The list is composed of five long-read-only assemblers^4,5,16,18,19^ and a hybrid assembler^31^ (MaSuRCA; Table 1). All benchmarked genome assemblies were generated by the developer of the respective assembler (Supplementary Table 3). In particular, the SHASTA assemblies were generated using an independent Nanopore dataset^19^, with a genome coverage of ~60×, and including at least 6× coverage of ultralong reads (>100 kb).

**Table 1:** WENGAN assemblies of the diploid NA12878, HG00733 and NA24385 genomes

		NA12878							HG00733			NA24385	
		WENGAN-M	WENGAN-A	WENGAN-D	MaSuRCA	CANU	WTDBG2	FLYE	WENGAN-D	SHASTA	FALCON	WENGAN-D	SHASTA
Assembly statistics	Contigs (≥50 kb)	490	425	**364**	1,111	798	934	797	**387**	649	826	**270**	660
	Total length (Mb)	2,779	2,780	2,823	2,876	2,824	2,701	2,898	2,812	2,802	2,893	2,871	2,819
	NG50 (Mb)	17.24	25.99	**35.31**	8.43	10.41	11.84	22.91	**32.35**	21.71	22.33	**50.59**	20.35
Structural quality	Reference covered (%)	94.22	94.30	95.25	**95.80**	95.05	91.70	95.56	95.12	94.98	**96.06**	**96.36**	95.61
	Duplication ratio	**1.002**	**1.002**	1.006	1.013	1.008	0.991	1.025	1.004	**1.002**	1.020	1.011	**1.002**
	Unaligned length (Mb)	5.21	**4.76**	8.63	24.68	9.42	32.13	21.29	6.96	**6.49**	15.41	10.54	**6.52**
	NGA50 (Mb)	11.82	14.34	**16.41**	5.69	7.12	7.38	12.36	**17.31**	12.99	14.61	**24.52**	14.32
	Longest alignment (Mb)	45.66	75.32	72.84	32.62	34.07	70.48	**78.99**	71.03	**78.22**	71.68	75.56	**75.65**
	Assembly errors	153	**91**	158	275	194	124	177	119	**107**	198	156	**126**
Computational resources	CPU hours (h)	**203**	725	589	20,000	~150,000	891	5,000	936	~**768**	20,000	963	~**768**
	Maximum RAM (Gb)	53	**45**	622	500	–	222	600	**644**	~966	–	**651**	~692
Consensus accuracy													
Indels	Short (Mb)	1.99	1.72	0.85	**0.56**	0.57	27.16	37.3/2.65	**0.64**	3.09	1.19	**0.62**	3.38
	Rate (bp)	1,381	1,592	3,252	**4,966**	4,828	98	74/1,047	**4,372**	899	2,323	**4,499**	828
	Medium (Mb)	0.45	0.43	**0.29**	0.38	0.35	1.85	2.43/0.74	**0.27**	0.7	0.28	**0.29**	0.73
	Rate (bp)	6,049	6,358	**9,447**	7,335	7,766	1,442	1,142/3,753	**10,161**	3,982	10,046	**9,608**	3,817
	Long (kb)	17.95	18.73	17.74	19.21	45.96	**12.64**	15.60/16.49	22.9	18.13	**17.65**	24.85	**23.05**
	Rate (kb)	153	146	157	145	60	**211**	178/169	121	153	**157**	113	**121**
	Per 100 kb	102	90	53	**47**	55	1,135	1,471/147	**36**	141	62	**39**	152
	100-mer completeness (%)	84.24	84.82	87.44	**87.54**	86.41	29.47	22.47/81.47	**87.45**	79.84	86.42	**88.53**	79.38
	Median QV	27.84	28.41	**31.02**	27.10	28.79	17.08	16.41/23.48	26.42	23.36	**27.30**	–	-
Gene completeness													
BUSCO	No. complete	3,884	3,893	**3,898**	3,866	3,882	1,974	2,268/3,680	**3,907**	3,788	3,874	**3,904**	3,752
	Percentage complete	94.64	94.86	**94.98**	94.20	94.59	48.10	55.26/89.67	**95.20**	92.30	94.40	**95.13**	91.42

Structural and consensus accuracy was determined as described in detail in the Methods (Assembly validation). All of the assemblies were built by the assembler developers. In particular, all of the NA12878 assemblies were generated using the Nanopore (rel5) plus Illumina data at the assembly or polishing steps (except WTDBG2). The CANU assembly was hybrid polished with NANOPOLISH ×2, RACON ×2 and PILON ×2. The FLYE assembly was hybrid polished with RACON ×2 and NTEDIT ×3 (Methods). The SHASTA assemblies were polished using only Nanopore reads with HELEN and MARGINPOLISH. The FALCON assembly was polished using only PacBio CLR reads with QUIVER. The WENGAN, MaSuRCA and WTDBG2 assemblies were not polished by external tools. The reported CPU time does not include the CPU time spent polishing the assembly with external tools. NG50 and NGA50 were computed using as the genome size the total chromosome lengths of GRCh38 (3.088 Gb). Assembly errors overlapping centromeric regions or SDs of GRCh38 were excluded from the analysis. The indels were called from aligned blocks ≥1 kb at average identity ≥99%, and were classified according to their length into short (1–2 bp), medium (3–50 bp) and long (>50 bp). The indel rate was computed by dividing the amount of assembly sequence aligned by the number of indels called on such alignments. The ‘indels per 100 kb’ was computed by QUAST from aligned blocks ≥0.5 kb with a minimum identity ≥80%. The 100-mer completeness is the fraction of distinct 100-mers in the GRCh38 reference (2.835 Gb) that are captured in the corresponding assembly. Consensus statistics before and after the polishing are included for the FLYE assembly. The best and worst performers on each assembly metric (rows) are highlighted in bold or underlined font, respectively.

For NA12878 (Table 1), WENGAN produced the most contiguous assemblies, with contig NG50 values of 17.24, 25.99 and 35.31 Mb for WENGAN-M, WENGAN-A and WENGAN-D, respectively. The best long-read assembler among the four evaluated, namely FLYE (NG50 22.91 Mb), is comparable to WENGAN-A (NG50 25.9 Mb), but is surpassed by WENGAN-D (NG50 35.3 Mb). All of the other evaluated assemblers are outperformed by any WENGAN mode (NG50 ≥ 17.24 Mb; Table 1 and Supplementary Fig. 9). Moreover, WENGAN increased the contiguity of the short-read-only assemblies by a factor of 1,833×, 2,014× and 388×, for MINIA3 (NG50 9.6 kb), ABYSS2 (NG50 12.9 kb) and DISCOVARdenovo (NG50 91 kb), respectively (Supplementary Table 7). The WENGAN-D assembly of HG00733 has the fewest gaps of any PacBio continuous long-read (CLR) assembly of a human genome, with more than half of the genome contained in contig sequences at least 32.3 Mb long (Table 1 and Supplementary Fig. 9), a substantial improvement in contiguity over the FALCON (NG50 22,3 Mb) and SHASTA (NG50 21.7 Mb) assemblies (Table 1). The WENGAN-D assembly of NA24385 (NG50 50.59 Mb) more than doubles the contiguity of SHASTA (NG50 20.35 Mb, Table 1), surpasses the contiguity of the GRCh37 reference (NG50 38.5 Mb) and matches the contiguity of the GRCh38 reference (Supplementary Fig. 9).

The structural quality was determined using QUAST^37^. The WENGAN assemblies cover up to 96.3% of the reference genome with few assembled sequences (<0.4%) unmapped to GRCh38 (Table 1, Reference covered (%) and Unaligned length), and the contigs have fewer duplicates than the contigs of its peers (except SHASTA; Table 1, Duplication ratio). The NGA50 (which corresponds to the NG50 corrected of assembly errors) of WENGAN-D (16.41–24.52 Mb) is the highest across the three assembled genomes (Table 1 and Supplementary Fig. 9). For NA12878, the NGA50 of WENGAN (11.8 Mb–16.41 Mb) almost doubles the ones of MaSuRCA (5.69 Mb), WTDBG2 (7.38 Mb) and CANU (7.12 Mb; Table 1). Moreover, WENGAN consistently showed a lower number of assembly errors than its peers (Table 1 and Supplementary Table 8). The only exception is SHASTA, a conservative assembler^19^, which has a lower number of assembly errors than WENGAN-D on the HG00733 (107 versus 119) and NA24385 (126 versus 156) genomes. However, WENGAN-D reaches higher NGA50 values than SHASTA and almost doubles the NGA50 achieved by SHASTA on the NA24385 genome (24.5 versus 14.3 Mb; Table 1 and Supplementary Fig. 9).

The consensus accuracy of genome assemblies was determined using different sequence analyses (Table 1 and Supplementary Table 9). The level of polishing of the assemblies goes from none to complete (Table 1), including examples of long-read-only (SHASTA and FALCON) and hybrid (short + long reads, CANU and FLYE) polishing (Table 1). For all three genomes, WENGAN reaches a higher consensus accuracy than unpolished or long-read-only polished assemblies (Table 1). In the NA12878 genome, the hybrid polished assemblies of CANU and MaSuRCA have better short-indel rates than the WENGAN assemblies, but WENGAN has better than or comparable medium- and long-indel rates (Table 1). Moreover, unlike long-read assemblers, the majority (≥73%) of the WENGAN consensus errors are located in the mate-edge consensus sequences (Supplementary Fig. 10), representing at most 10% of the WENGAN assembled sequence. The 100-mer analysis reveals that the WENGAN assemblies contain at least 84.5% of the 100-mers of the reference (Table 1). The BUSCO gene completeness of the WENGAN assemblies ranges from 94.62% to 95.20%, which is higher than the result of any other evaluated assembler and reflects the high consensus quality and contiguity of the WENGAN assemblies (Table 1). Hybrid polishing of the FLYE assembly consumed 755 CPU hours (Supplementary Table 10). While the hybrid polishing removed millions of consensus errors (Table 1 and Supplementary Table 10), and increased the median quality value and the BUSCO gene completeness (to 23.39 and 89.7%), the hybrid-polished FLYE assembly still has a lower quality than any of the unpolished WENGAN assemblies (Table 1).

We analyzed how hard-to-assemble regions are resolved on these diploid human genomes (Supplementary Figs. 11a and 12). WENGAN with ultralong reads spans the MHC region with fewer than four contigs (Supplementary Fig. 11b). The top performers, namely CANU (NA12878), FALCON (HG00733) and WENGAN-D (NA243875), solve the MHC region in a single contig achieving NGA50 values ≥3.5 Mb (Supplementary Fig. 11b). However, all of the evaluated assemblers produce a mix of haplotypes, and therefore subsequent phasing must be performed to fully solve the MHC region^29^. Regarding SDs (Supplementary Fig. 12), WENGAN-M and WENGAN-A resolve over 41 Mb (~6 Mb of SDs >100 kb), which is better than WTDBG2 (17 Mb) and comparable to SHASTA ($\bar x = 42{\mathrm{Mb}}$; Supplementary Fig. 12). WENGAN-D resolves more SD sequences with ultralong Nanopore reads (56.09–60.12 Mb) and matches the top performer CANU on NA12878 (56.09 versus 56.98 Mb). With PacBio reads, the FALCON assembler resolves 6.4 Mb more SD sequences than WENGAN-D (Supplementary Fig. 12). The SD analysis of these three diploid samples shows that WENGAN-A and WENGAN-M are more conservative than WENGAN-D for SD assembly, and that WENGAN-D is comparable to the top performers (FLYE and CANU), while achieving a lower rate of assembly errors (Table 1, Fig. 2b and Supplementary Fig. 12).

In terms of computational resources, the WENGAN assemblies consumed less than 1,000 CPU hours (Table 1 and Supplementary Table 8, maximum elapsed time of 45 h). WENGAN-M, the fastest WENGAN mode based on MINIA3, consumed ~738 times less CPU hours than CANU (203 versus ~150,000 CPUh; Table 1) and required only 53 Gb of RAM to complete the assembly (Table 1).

Collectively, the benchmark results demonstrate that WENGAN is the only genome assembler evaluated that optimizes all of the 1–2–3 de novo assembly goals, namely, contiguity, consensus accuracy and computational resources.

### WENGAN is effective at low long-read coverage

**Fig. 4:**
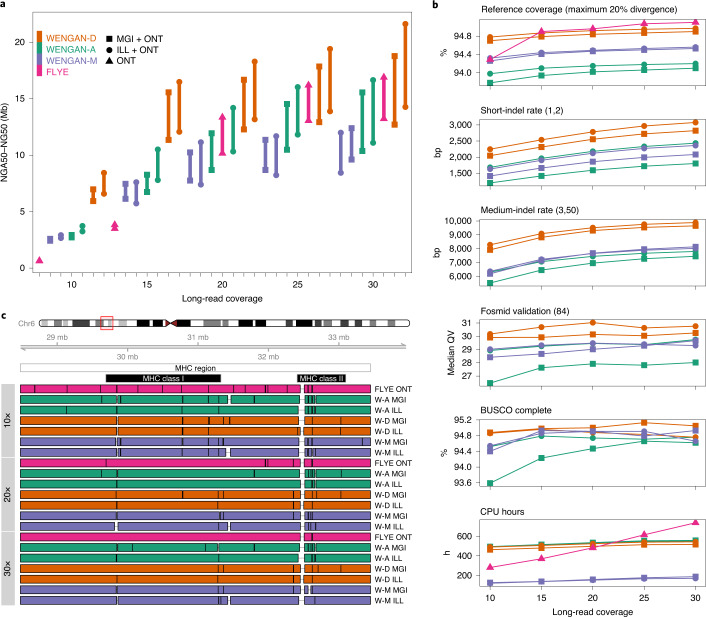
De novo genome assemblies of NA12878 when varying the long-read coverage and the short-read technology. **a**, The de novo assemblies were sorted by NG50 at each long-read coverage (lolliplot). We computed the NGA50 (which corresponds to the NG50 corrected of assembly errors) of each assembly using QUAST (see Methods). **b**, The consensus quality (see Methods) of each genome assembly and the CPU hours required for the assembly. **c**, The WENGAN (W-*X*) and FLYE assemblies of the complex MHC region located in Chr6: 28,477,797–33,448,354 (4.97 Mb). The MHC sequence was aligned to the genome assemblies and the aligned blocks ≥30 kb with a minimum identity of 95% were kept. The alignment breakpoints (vertical black lines) indicate a contig switch, an alignment error or a gap in the assembly. The assemblies of the MHC region are displayed in tracks by long-read coverage. Source data

We investigated the required long-read coverage to produce de novo assemblies with an NG50 of at least 10 Mb. Moreover, we assessed the suitability of the MGI sequencing technology^11^ (MGISEQ-2000) as an alternative to Illumina SBS^10^ for hybrid assembly using matched short-read genomic data. We sequenced the NA12878 human cell line using the short-read sequencers NovaSeq 6000 (ref. ^10^) and MGISEQ-2000 (ref. ^11^) as well as the long-read sequencer ONT PromethION^13^ (Methods). We generated a total of 548.2 million pair-end reads (2 × 150 bp) of sequence (53.06×) from both short-read sequencers (Supplementary Table 2). Furthermore, three flow cells of PromethION produced a total of 10.4 million reads (40×) with a N50 of 17.18 kb (Supplementary Table 1). We randomly subsampled the long-read data from 10× to 30× genome coverage in increasing batches of 5×. The N50 was nearly identical for all of the long-read subsamples (N50 = 19.6 kb; Supplementary Table 11). WENGAN and the best long-read assembler among those evaluated, namely FLYE (v.2.5), were used to build hybrid and long-read assemblies for each subsample (Fig. 4 and Supplementary Table 12).

A major increase in contiguity for WENGAN was observed when going from 10× to 15× long-read coverage (Fig. 4a and Supplementary Table 12). In particular, we observed an NG50 increase from 2.5, 2.9 and 6.9 Mb to 7.4, 8.2 and 15.5 Mb for WENGAN-M, WENGAN-A and WENGAN-D, respectively. At shallow long-read coverage (10–15×), FLYE is outperformed by all WENGAN modes. Over 20× coverage, FLYE outperforms WENGAN-M and is comparable in contiguity to WENGAN-A (Fig. 4). Notably, WENGAN-D using 15× long-read coverage leads to an NG50 of 15 Mb, which FLYE can reach only at 30× long-read coverage (Fig. 4a).

All assemblies generated by WENGAN cover more than 93.8% of the reference genome at any long-read coverage (Fig. 4b). As expected, FLYE achieves its highest consensus quality at 30× long-read coverage (maximum QV = 21.08; Supplementary Table 13). Polishing FLYE with long and short (NovaSeq) reads increased its median consensus quality to QV = 27.21 (Supplementary Table 14). Almost all WENGAN assemblies achieve a higher consensus quality than the polished FLYE assembly (minimum WENGAN QV = 27.67 excluding WENGAN-A-MGI-10×; Fig. 4b and Supplementary Tables 12 and 13).

The contiguity and consensus quality of the WENGAN assemblies vary more as a function of WENGAN’s mode than with the type of short-read data used (Fig. 4a,b). Indeed, under the same WENGAN mode, the largest difference in contiguity between the short-read technologies of Illumina and MGI is NG50 = 2.8 Mb (WENGAN-D at 30×, Fig. 4a) and their consensus quality is almost identical (Fig. 4b). WENGAN-M required a maximum of 187 CPU hours (maximum elapsed time < 18.1 h on 20 CPUs) and 44 Gb of RAM to complete the assemblies (Fig. 4b and Supplementary Table 12). To our knowledge, this is the first time that a genome assembler reaches a contiguity of 10 Mb and consensus quality of QV 29.4 on such minimal and accessible sequencing and computing resources.

We checked the assemblies of FLYE and WENGAN to determine whether they solved the 4.97 Mb MHC region (Fig. 4c). The WENGAN assemblies at low coverage (≤20×) reach higher NGA50 than the FLYE assemblies (Fig. 4c and Supplementary Fig. 13). However, FLYE over 25× coverage assembles the MHC region in fewer than two contigs with a NGA50 of 4 Mb (Fig. 4c and Supplementary Fig. 13).

In summary, we demonstrated that WENGAN reduces the computational resources and the long-read coverage required for assembling a human genome. WENGAN produced a high-quality assembly with NG50 > 10 Mb (QV > 29) by combining 20× long-read coverage with 50× short-read coverage using less than one day of computing time on a low-end server (20 cores, ≤50 Gb RAM).

## Discussion

We have demonstrated that WENGAN is the only genome assembler that optimizes the three main goals of de novo assembly algorithms, namely, contiguity, consensus accuracy and computational resources. Furthermore, WENGAN is effective at shallow long-read coverage (≥15×), and in combination with ultralong reads generated de novo assemblies that surpass the contiguity of the human reference genome GRCh38. We introduced a hybrid assembly combining accurate PacBio/HiFi reads with ultralong Nanopore reads and achieved an assembly quality that rivals the quality of the assembly generated by the T2T consortium (v.0.7)^30^. Additionally, we observed no notable difference in assembly quality between using the short-read platforms Illumina NovaSeq 6000 (ref. ^10^) or MGISEQ-2000 (ref. ^11^) for hybrid assembly with WENGAN. Moreover, WENGAN produces high-quality assemblies with any combination of short-read (NovaSeq or MGISEQ-2000) and long-read (ONT MinION/PromethION or PacBio Sequel I) technologies.

Unlike current long-read assemblers, WENGAN generates functional and ready-to-use genome reconstructions. The consensus quality benchmark demonstrated that short-read polishing remains mandatory for assemblies generated from Nanopore and PacBio CLR reads (Table 1 and Supplementary Tables 3, 9 and 13). Although PacBio’s HiFi reads represent an option that mitigates the post-assembly polishing and, in combination with ultralong Nanopore reads, generates assemblies with the highest contiguity, this comes at a reduced throughput (~10 CLR reads to generate 1 HiFi read) and substantially increased computational resources^26,28^. We found that hybrid WENGAN assemblies provide a computationally efficient solution for human genome assembly, producing, at the same time, highly competitive assembly contiguity and quality.

Previous genome assemblers cannot cope with the high throughput of a long-read and a short-read sequencer. Although other long-read-only assemblers may have a similar real-time execution^19^ (one day), they require less accessible computational resources and more long-read coverage, and process half the data compared with WENGAN. Still, our analyses of hard-to-assemble regions demonstrated that further algorithmic improvements are necessary for all examined assemblers. Even though we have centered our analysis on human genomes, WENGAN also achieves high assembly quality of non-human genomes (complete BUSCO genes ≥95%; Supplementary Table 15). Moreover, the WENGAN approach also provides a natural framework to combine long-read with linked-read data, and/or Sanger-size short reads^39^, and/or optical maps (BIONANO), which may lead to the assembly of ‘telomere-to-telomere’ scaffolds without the need for extra polishing and finishing methods. Therefore, WENGAN should facilitate the democratization of de novo assembly of human genomes, enabling high-quality genome assembly for many applications. The WENGAN assembler is available at GitHub (https://github.com/adigenova/wengan) and Code Ocean (10.24433/CO.9469612.v1).

## Methods

### The WENGAN algorithm

#### Short-read assembly

WENGAN can employ MINIA3 (ref. ^6^), ABYSS2 (ref. ^7^) or DISCOVARdenovo^8^ as the de Bruijn graph-based short-read assembler. All three short-read assemblers are able to assemble a human genome in less than a day. MINIA3 and ABYSS2 were intended for low-memory assembly of large genomes. They are able to assemble human genomes using less than 40 Gb of RAM^6,7^. MINIA3 is the fastest method, consuming less than 77 CPU hours to complete a human genome assembly (Supplementary Table 7). Its speed comes from the novel unipath algorithm BCALM2 (ref. ^40^) that uses minimizers^20^ to compress quickly and with low memory the de Bruijn graph^40^. MINIA3 can be used iteratively to implement a multi-*k*-mer assembly approach. We used *k*-mer sizes of 41, 81 and 121 in all of the WENGAN-M assemblies described (Supplementary Table 7). ABYSS2 uses a Bloom filter and rolling hash functions as the main techniques to implement the de Bruijn graph-based assembly^7^. After filling the Bloom filter, ABYSS2 selects solid reads (that is, reads composed only of solid *k*-mers, namely those for which frequency(*k*) > 2) as seeds to create the unipaths. These are extended left and right by navigating in the de Bruijn graph until a branching vertex or a dead end is encountered. In our benchmark tests, ABYSS2 required on average 481 CPU hours to assemble a human genome (Supplementary Table 7). All of the ABYSS2 assemblies were run using a Bloom filter size of 40 Gb (*B* = 40 G), four hash functions (*H* = 4), solid *k*-mers with a minimum frequency of 3 (kc = 3), *k*-mer size 96, and only until the contig step. DISCOVARdenovo is a more specialized algorithm designed to assemble a single PCR-free paired-end Illumina library containing ≥150-bp reads. DISCOVARdenovo is greedier in terms of memory than MINIA3 and ABYSS2. We observed a memory peak of 650 Gb in our human assemblies (Supplementary Table 7). However, DISCOVARdenovo better leverages the pair-end information and therefore produces the most contiguous short-read assemblies of all three tested assemblers (average contig NG50 69 kb; Supplementary Table 7). All of the selected short-read assemblers refine the constructed de Bruijn graph by removing sequencing errors and collapsing the genomic variants (single-nucleotide polymorphisms and indels) to produce accurate consensus contigs^6–8^.

#### Pair-end pseudo-alignment as a building block for genome assembly

In the same way as *k*-mers are the elemental building blocks of de Bruijn graph assemblers, WENGAN relies on pair-end pseudo-alignments as the elemental building blocks for the de novo assembly. We recently introduced an alignment-free method called FAST-SG^32^ that uses unique *k*-mers to compute a pseudo-alignment of pair-end reads from long- or short-read technologies. Here, we present its successor, which we called FASTMIN-SG, which implements the same ideas as FAST-SG but using minimizers^20^ and chaining with the MINIMAP2 application programming interface^41^. The uniqueness of the pseudo-alignment is now determined using the MINIMAP2 mapping quality score, which gives a higher score to a primary chain when its best secondary chain has a weak pseudo-alignment.

To perform a pseudo-alignment of pair ends from short-read sequencing technologies, we use (10,21)-minimizers for querying and indexing. We discard pair-end pseudo-alignments when one of the mates has a mapping quality score ≤30 or covers ≤50% of the read bases. For mapping synthetic pair ends extracted from long-read technologies, we use (5,20)-minimizers and a read length of 250 bp. A synthetic pair end is a fragment of length *d* for which we have access to the long read of origin, the position of the fragment in the long read and the inner long-read sequence between both mates of the synthetic fragment. All of the synthetic fragments are extracted from the long reads using a moving window of 150 bp in forward–reverse orientation. We create a spectrum of synthetic mate-pair libraries (Supplementary Fig. 3) by extracting pair ends at different distances. The range of distances depends on the long-read lengths but go from 0.5 kb to a maximum of 500 kb with ultralong Nanopore reads. For noisy PacBio reads, we use homopolymer-compressed *k*-mers^41^ for indexing and querying the synthetic pair ends. We discard synthetic pair-end alignments when one of the mates has a mapping quality score ≤40 or covers ≤65% of the synthetic read bases. The information associated with the long read of each synthetic pair is stored in the read names for computing approximate long-read alignments later. FASTMIN-SG, like MINIMAP2, uses presets to modify multiple parameters, thus simplifying its usability. Currently, it has presets for raw PacBio reads (pacraw), HiFi reads (pacccs), raw (ontraw) and ultralong (ontlon) Nanopore reads, and pair ends (shortr) from short-read technologies (supporting Illumina or MGI). The pseudo-alignments are reported in SAM format.

#### Detection and splitting of chimeric short-read contigs

The de Bruijn graph is complex around repeat sequences, and short-read assemblers can choose wrong paths while traversing such complex regions, thus leading to chimeric contigs (Supplementary Fig. 1). To detect potential chimeric contigs not supported by the short reads, we map the pair-end reads back to the assembled short-read contigs using FASTMIN-SG (preset shortr). From the pair-end pseudo-alignments, we infer the average $\bar x$ and standard deviation *σ* of the insert-size distribution of the genomic library. Then, pair ends mapped within contigs at the expected orientation and distance $\left( {\left[ {\bar x - 2.5\sigma ,\bar x + 2.5\sigma } \right]} \right)$ are transformed into physical fragments. For each contig, we create an array of length equal to the contig length, and the contig fragments are used to increase the physical coverage of the contig bases. We then scan the physical coverage array base-by-base to detect low-quality intervals (LQIs) that have a fragment coverage below a minimum depth threshold (def: 7). LQIs are classified according to their contig location as internal, start, end or whole. Finally, contigs are trimmed/split at the boundaries of the LQIs.

#### The SSG

We build on the work of Huson et al.^33^ to extend the scaffolding graph formulation and create the SSG. In brief, the contig scaffolding problem was defined by Huson et al.^33^ as the determination of an order and orientation of a set of contigs that maximize the amount of satisfied mate-pair links. The scaffolding graph *G* = (*V*, *E*), with vertex set *V* and edge set *E*, is a weighted, undirected multi-graph, without self-loops^33^. Each contig *C*_*i*_ is modeled by two vertices (*v*, *w*) and an undirected contig edge (*e*). The length of *e* is set to the contig length *l*(*C*_*i*_). The contig orientation is represented by associating each of the contig ends to one of the two vertices (that is, tail(*C*_*i*_) = *v* and head(*C*_*i*_) = *w*). Then traversing from tail(*C*_*i*_)→head(*C*_*i*_) or head(*C*_*i*_)→tail(*C*_*i*_) implies forward or reverse contig orientation, respectively. Now, consider a pair of mate reads *f* and *r* originated from a synthetic mate-pair library with mean insert size $\bar x$, standard deviation *σ* and orientation forward–reverse that uniquely matches two different contigs *C*_*i*_ and *C*_*j*_. The uniquely mapped mate pair induces a relative orientation and approximate distance between the two contigs. Such information is represented by adding a mate edge *e* into the graph. The length of the mate edge *e* is computed by subtracting from the expected mate-pair distance $\left( {\bar x} \right)$ the amount of overlap that each contig has with the mate pair considering the read mapping orientations: $l\left( e \right)= \bar x - \left( {l\left( {C_i} \right) - {\rm{ pos}}_{C_i}\left( f \right)} \right) - \left( {l\left( {C_j} \right) - {\rm{ pos}}_{C_j}\left( r \right)} \right)$. Moreover, the standard deviation *σ*(*e*) of each mate edge *e* is set equal to the standard deviation of the synthetic mate-pair library. If there is more than one mate edge *e* between the same ends of two contigs *C*_*i*_ and *C*_*j*_, we can bundle^33^ the mate edge *e* by computing from the set of mate edges *e*_1_, *e*_2_,…*e*_*n*_ the length of *e* as *l*(*e*) := *p*/*q* and its deviation as $\sigma \left( e \right) = \sqrt {1/q}$, where $p = {\sum} {\frac{{l(e_i)}}{{\sigma (e_i)^2}}}$ and $q = {\sum} {\frac{1}{{\sigma (e_i)^2}}}$ (ref. ^33^). Additionally, the weight *w*(*e*) of a bundled mate edge *e* is set to $\mathop {\sum}\nolimits_{i = 1}^k {w(e_i)}$, and otherwise to 1.

The SSG is an edge-bundled scaffolding graph *G* = (*V*, *E*), built from a spectrum of synthetic mate-pair libraries, where there is an edge-labeling function (*F*) that maps the long reads to the edges through the synthetic mate-pair pseudo-alignments.

#### Computing approximate long-read overlaps with the SSG

As the SSG is built from a spectrum of synthetic mate-pair libraries (that is, 1 kb to 10 kb; 3 in Fig. 1), it contains mate edges from the short (1 kb) to the long (10 kb) range of connectivity (that is, *e*1, *e*4; 5 in Fig. 1). Now, consider a mate edge *e* from *v* to *w* that are also connected by a transitive path *P* = (*m*_1_, *c*_1_, *m*_2_,…*m*_*k*_) of mate edges (*m*_1_, *m*_2_,…), contig edges (*c*_1_, *c*_2_,…) and long-read labels *F*(*P*) = (*F*(*m*_1_), *F*(*c*_1_), *F*(*m*_2_),…*F*(*m*_*k*_)). We can compute the path length *l*(*P*) and its standard deviation *σ*(*P*) as follows^33^: $l\left( P \right) = {\sum} {l\left( {m_i} \right) + {\sum} l (C_i)}$ and $\sigma \left( p \right) = \sqrt {{\sum} {\sigma (m_i)^2} }$. A mate edge *e* from *v* to *w* (that is, *e*4; 5 in Fig. 1) can be transitively reduced on the path *P* (that is, *P*2 = (tail(*c*_2_), *e*_3_, *c*_3_, *e*_5_, head(*c*_4_)); 6 in Fig. 1) if *e* and *P* have similar lengths and the long-read labels of *e* are coherent with every edge *e*_*i*_ of *P*: |*l*(*e*) − *l*(*P*)| ≤ 4max(*σ*(*e*), *σ*(*P*)) and *F*(*e*) ⊂ *F*(*e*_*i*_) ∀ *e*_*i*_ ∈ *P*. If this is the case, then the transitive path *P* (that is, *P*2) is long-read coherent with the mate edge *e* (that is, *e*_*i*_) and represents an approximate overlap of length *l*(*P*) among all of the long reads composing the mate edge *e* (*F*(*e*)). We store the overlap information by removing *e* (that is, *e*4) from the SSG and incrementing the weight of every mate edge *m*_*i*_ in *P* by *w*(*e*) (that is, *w*(*e*_3_) = *w*(*e*_3_) + *w*(*e*_4_) and *w*(*e*_5_) = *w*(*e*_5_) + *w*(*e*_4_); 6 in Fig. 1). Before starting the computation of approximate long-read overlaps, the repetitive contig edges are masked, the mate edges are sorted by ascending length *l*(*e*) and the set of biconnected components of the SSG is computed. The masking of repetitive contig edges is performed by estimating the average coverage of unique genomic regions using as a proxy the longest (10%), all likely to be single-copy, short-read contigs ($\bar{u}$). Contig edges with an average coverage $\overline {cx} > 1.5 \times \bar u$ are masked by default. This repeat masking procedure is similar to but simpler than the A-statistic and threshold (~1.44) introduced by Myers^3^. Transitive long-read-coherent path search takes place inside each biconnected component. In practice, we use a depth-first search algorithm to enumerate all of the long-read-coherent paths of a given mate edge *e*. At each edge extension, we extend the path only if the new added edge is long-read coherent with the given mate edge *e* (*F*(*e*) ⊂ *F*(*e*_*i*_)). We stop searching when the size of a partial path *P* is larger than 80 vertices or its length is longer than expected (*l*(*P*) > *l*(*e*) and |*l*(*e*) − *l*(*P*)| > 4max(*σ*(*e*), *σ*(*P*))). If there is more than one long-read-coherent path, we choose the path having the maximum number of hits from the long reads supporting the given mate edge *e*. For very long mate edges (*l*(*e*) ≥ 100 kb), we stop searching if we find more than 100 long-read-coherent paths. All of the selected long-read-coherent paths are stored in a path database for later use.

The final SSG graph is created by performing first bundling and then transitive reduction (approximate long-read overlaps) of mate edges. From now on, we will refer to this simply as the reduced SSG.

#### Generation of the assembly backbone with the SSG

Computation of approximate long-read overlaps allows one to solve the scaffolding problem using all of the synthetic mate-pair libraries simultaneously. Given the reduced SSG, our goal is to determine an optimal set of vertex disjoint paths covering all of the contig edges with a maximum total weight of the mate edges. As this optimization problem is NP-hard (non-deterministic polynomial-time hard), we use Edmond’s maximum weighted matching approximation algorithm that guarantees to find an optimal solution with a worst-case performance ratio *r* = *W*(*S*)/*W*(*G*) > 2/3 (ref. ^34^). The matching algorithm implementation is based on an extensive use of priority queues, leading to an *Ο*(*VE*log[*V*]) time complexity^42,43^. All of the contig edges, as well as the mate edges associated with repetitive contigs or having a weight smaller than 5, are masked during the matching cover step. After computing the matching cover, all of the contig edges are added to the matching cover solution and we use a depth-first search approach to detect simple cycles. If such cycles are found, the set of biconnected components of the graph is computed and simple cycles are destroyed by removing the mate edge of lowest weight in each biconnected component. In practice, the matching cover solutions contain few cycles (<10 on human genomes) and we observed performance ratios higher than *r* > 0.8. The set of optimal simple paths (lines or scaffolds) is what we call the assembly backbone.

#### Validation of the assembly backbone with the SSG

We validate the assembly backbone using the physical genomic coverage obtained from the computation of the approximate long-read overlaps. The key idea is to identify suspicious mate edges *e* (corresponding to potentially incorrect joins) not supported/covered by long-read overlaps longer than *O* (by default *O* ≥ 20 kb). We first assign genomic coordinates to each line *L*_*i*_ = (*c*_1_, *m*_2_,…*m*_*k*−1_, *c*_*k*_) from 1 to *l*(*L*_*i*_), taking into account the orientation of the contig edges and the ordering and distance provided by the matched mate edges. In a second step, all of the contig edges are converted into physical genomic fragments as well as the mate edges spanned by long reads longer than *O*. In a third step, if the vertices *v*, *w* of a reduced mate edge *e* belong to the same line *L*_*i*_ and the length of *l*(*e*) is longer than *O*, we create a new simple path pf that goes from *v* to *w* in the line *L*_*i*_. The new simple path pf is converted into a physical genomic fragment *f* only if the length of pf is similar to the length of the reduced mate edge *e*; that is, if |*l*(*e*) − *l*(pf)| ≤ 4max(*σ*(*e*), *σ*(pf)). If that is the case, the simple path pf increases the physical genomic coverage of the line *L*_*i*_. In a fourth step, once the physical genomic coverage of all the lines *L*_*i*_ has been computed, we look for all of the intervals inside a scaffold having a lack of physical coverage at the mate-edge locations and we tag such mate edges as potentially erroneous joins. A line *L*_*i*_ is split at potential error joins only if the number of long reads supporting the suspicious mate edge *e* is less than mlr (default mlr *≤* 4). In practice, we observe that the physical path coverage of human assemblies is around 20× (30× long-read coverage); thus, usually fewer than 200 mate edges are removed.

#### Gap filling with the SSG

A property of the SSG is that all of the mate edges are spanned by at least one long read. Therefore, after construction and validation of the assembly backbone, we proceed to create a consensus sequence for each of the matched mate edges. We start by ordering the lines by decreasing length, which imposes a global order to the mate edges and consequently to the long-read sequences. For each mate edge *e*, we select the *N* best long reads (default: 20) spanning *e*. The long-read selection is carried out by counting, with the edge-labeling function *F*(*e*), the number of synthetic mate pairs contributed by the long-read *l*_*i*_ to compose the mate edge *e*. This means that, the more synthetic mate pairs are contributed by long-read *l*_*i*_, the greater is the confidence that the long-read *l*_*i*_ spans *e*. All of the selected long-read sequences are sorted according to the mate-edge order using an external merge sort algorithm to create a long-read sequence database. Following the long-read database creation, we build a consensus sequence for each mate edge using the partial order alignment graph^35^. For each mate edge, we select the long read contributing the most synthetic mate pairs as the consensus template; then the remaining long reads spanning *e* are aligned to the template using a fast implementation of Myers’s bit-vector algorithm^36^. The long-read alignments are scanned to partition the long reads into non-overlapping windows of size *w* (by default 500 bp) on the template sequence. The long-read chunks that have an average identity lower than 65% are removed from the corresponding windows. The purpose is to use high-quality alignments to build the template consensus. For each window *w*, we call the consensus sequence using an SIMD-accelerated (single instruction multiple data) implementation of the partial order alignment graph^17^. The mate-edge consensus is built by joining the window sequences. Finally, the corresponding contig ends are aligned (using once again Myers’s bit-vector algorithm) to the mate-edge consensus sequence to determine the correct mate-edge sequence boundaries, thus filling the gap between the two contig edges.

#### Polishing with the SSG

As not all of the contig edges are part of the assembly backbone (as is the case for the contig edges related to repeats or short sequences), we can use them to improve the consensus base accuracy of the mate-edge sequences. To this end, we use two polishing strategies, one based on the SSG and a second based on pairwise alignments. The graph polisher uses the reduced SSG to find transitive long-read-coherent paths as before, but masking the contig edges composing the assembly backbone. Since now we navigate on more complex parts of the SSG (unmasked repeat sequences), we limit the path search to a maximum of 5 million iterations on each mate edge. Once a long-read-coherent path has been found, we align the contig edges (with the proper orientation) to the mate-edge sequence using Myers’s bit-vector algorithm^36^. Then, the alignments are trimmed as a function of the average long-read depth of the mate-edge consensus sequence. We thus expect a minimum identity between 80% and 99% when the average long-read depth of the consensus sequence is between 1 and 20, respectively. If a contig edge maps with an identity higher than the expected and the alignment covers at least 75% of the contig edge, we replace the corresponding mate-edge-aligned sequence with the contig-edge-aligned sequence, thus polishing the mate-edge sequence. The alignment polisher searches for matches between the singleton contig edges and all of the mate-edge consensus sequences. In brief, we first index all of the mate-edge consensus sequences using (5,17)-minimizers^20^. Minimizers are stored in a hash table and the ones having a frequency higher than 1,000 are excluded. The (5,17)-minimizers of the contig edges are scanned on the mate-edge sequence index to collect high-scoring segment pairs or exact (5,17)-minimizer matches. High-scoring segment pairs are sorted by mate edges and hits are identified by finding the longest strictly increasing subsequence (co-linear chain) between the contig and the mate edges. After collecting all of the hits, we use a greedy algorithm to determine a layout of contig-edge hits along the mate-edge sequence. The greedy algorithm starts by sorting the contig-edge hits by number of minimizer matches and then adds the hits to the layout only if there is no overlap with a previously added hit. We then proceed as in the graph polisher to align and polish the mate-edge sequence using the best-hit layout. Finally, WENGAN outputs the sequence of each line plus the sequence of contig edges (>5 kb) not used in the polishing steps.

### WENGAN assemblies of CHM13

The WENGAN (HiFi + UL) assembly of the haploid CHM13 genome was generated using the WENGAN-M mode. The PacBio/HiFi reads were assembled with MINIA3 using an iterative multi-*k*-mer approach with the following *k*-mer sizes: 41, 81, 121, 161, 201, 251, 301 and 351. The PacBio/HiFi reads were then included in all of the subsequent WENGAN-M steps (Fig. 1). The WENGAN (ILL + UL) assembly was generated using the WENGAN-D mode. The specific commands to reproduce both WENGAN assemblies are provided in Supplementary Subsection 1.2.

### Assembly validation

Genome assemblies generated by WENGAN and other assemblers were assessed by whole-genome alignment to the human reference genome using the QUAST^37^ (v.5.0.2) tool. QUAST was run with the options –large –min-identity 80 –fragmented using the GRCh38 (patch 19) reference (autosomes plus X and Y). We also ran a QUAST analysis using as a reference the curated CHM13 assembly (chm13.draft_v0.7, 2.9384 Gb) generated by the T2T consortium^30^ for all of the CHM13 assemblies (Supplementary Table 4). Several assembly metrics (that is, NG50, NGA50, longest alignment block, indels per 100 kb, genome fraction and others) were collected from the QUAST report. QUAST assembly errors overlapping centromeric regions or SDs annotated in GRCh38 were excluded from the analysis using the script and annotation files provided by Shafin et al.^19^ (quast_sv_extractor.py -s empty -d GRCh38_masked_regions.bed -c centromeres.bed -q quast-all_alignments.tsv). The procedure masked a total of 610 Mb of the GRCh38 reference. Assembly errors before and after the masking of highly repetitive regions are reported. The consensus quality was determined by computing a more stringent alignment allowing a maximum of 1% divergence using the MINIMAP2 (ref. ^41^) program (MINIMAP2 options: cxasm10 –cs -r2k), and then contig-to-reference alignments longer than 1 kb were scanned by PAFTOOLS (option call −l1000 −L1000) to call single-nucleotide variants, insertions and deletions. Additionally, we used the 100-mer completeness analysis to assess with an alignment-free method the consensus quality of the genome assemblies using the KMC^44^ *k*-mer counter (v.3.1.0). The GRCh38 (patch 19) reference genome has a total of 2,835,070,131 distinct 100-mers and those were intersected with the 100-mers of the genome assemblies using the KMC_TOOLS utility (option intersect -ci1 -cx1000). The gene completeness of the genome assemblies was assessed with the BUSCO^45^ program (v.3.0.2) using the MAMMALIA ODB9 gene set (4,104 BUSCO groups). The single plus duplicated complete BUSCO gene counts are reported. The consensus quality of the genome assemblies was determined by aligning orthogonal BAC or fosmid sequence data (Supplementary Table 16). The statistics were computed considering fully resolved BAC/fosmid alone. The BAC/fosmid consensus quality analysis was performed using the BACVALIDATION tool (https://github.com/skoren/bacValidation). The amount of SD resolved by the genome assemblies of CHM13, HG00733, NA12878 and NA24385 was determined using SEGDUPPLOTS^38^ (https://github.com/mvollger/segDupPlots). SEGDUPPLOTS aligns the assembled contigs to GRCh38 and considers an SD as resolved when the aligned contig extends the SD flanking sequences by at least 50 kb. The sequence of the T2T-X chromosome was repeat-masked with the REPEATMASKER program (v.4.1.0, search engine: HMMER v.3.2.1, options: -species human -gff -xm) using the DFAM (v.3.1) database. The contigs of the CHM13 assemblies were anchored to the T2T-X chromosome using MASHMAP (v.2.0) and then masked with REPEATMASKER using the aforementioned options. Finally, the WENGAN assemblies of CHM13 were validated and scaffolded using the hybridScaffold.pl program (BIONANO Solve3.4_06042019a) (with the options -c hybridScaffold_DLE1_config.xml -B 2 -N 2) and the BIONANO map was assembled by the T2T consortium^30^.

### Hybrid polishing of FLYE assemblies

We polished the FLYE assemblies of NA12878 using the same sequencing reads employed in the WENGAN assemblies. We used two rounds of long-read polishing with RACON^17^ followed by three rounds of short-read polishing with NTEDIT^24^. The commands executed as well as the consensus quality improvement after each round of polishing are provided in Supplementary Tables 10 and 14.

### Genome sequencing of NA12878

The genomic DNA from the GM12878 human cell line was purchased from the Coriell Institute (catalog no. NA12878, RRID:CVCL_7526).

#### MGI sequencing

Library preparation for the NA12878 sample was performed with the MGIEasy DNA Library Prep Kit V1.1 (MGI, 940-200022-00) following the manufacturer’s instructions. Briefly, 1 μg genomic DNA at a concentration of 12.5 ng μl^−1^ was fragmented with an E220 Covaris program optimized to yield fragments of 450 bp in average length. A double-sized selection was performed with AMPure XP beads (Beckman Coulter) at 0.52× ratio followed by a 0.15× ratio as recommended by MGI. A total of 50 ng fragmented DNA was used for the end repair and A-tailing reaction following the manufacturer’s instructions. A set of adapters with 8 barcodes were ligated to the repaired DNA for 1 h at 23 °C. After purification with AMPure XP beads (Beckman Coulter) at a 0.5× ratio, the DNA was subjected to PCR enrichment following the manufacturer’s instructions. A total of 330 ng PCR product was hybridized with the Split Oligo (MGI, 940-200022-00) for the circularization step followed by digestion. Circularized single-stranded DNA (ssDNA) was purified with Library Purification Beads (MGI, 940-200022-00) and quantified with an ssDNA assay on a Qubit 3 fluorometer (Thermo Fisher). For the linear amplification to generate DNA nanoballs (DNBs), 75 fmol circularized ssDNA was used. The DNB library was loaded in a single lane and sequenced on an MGISEQ-2000 instrument with a paired-end modus and read length of 150 bp with the MGISEQ-2000RS High-Throughput Sequencing Set PE150 (MGI, 1000003981) according to the manufacturer’s instructions.

#### Illumina sequencing

The library was prepared using the TruSeq DNA PCR-Free Library Prep kit (Illumina, FC-121-3001) following the TruSeq DNA PCR-free reference guide (Illumina, 1000000039279v00). Briefly, 1 μg genomic DNA was used for fragmentation on an E220 Covaris to yield insert sizes of 350 bp. The DNA was end-repaired, adenylated and subjected to adapter ligation as described in the reference guide. The library was quantified using the KAPA Lib Quantification Kit (Roche, LB3111) and the double-stranded DNA (dsDNA) HS (high sensitivity) assay (Qubit). The average fragment size was estimated with an HS DNA kit (Agilent) on a 2100 Bioanalyzer (Agilent). An S2 flow cell loaded with 2.2 nM library was processed on a NovaSeq 6000 instrument to generate 2 × 150 paired-end reads.

#### Nanopore sequencing

Three flow cells were run with the sample NA12878. One flow cell was loaded with a library prepared from unsheared genomic DNA. For the additional two sequencing runs, 14 μg NA12878 genomic DNA was mechanically sheared with Megaruptor 3 (Diagenode) (at a concentration of 70 ng μl^−1^ in a volume of 200 μl) with the manufacturer’s recommended speed to get sheared DNA with an average fragment length of 30 kb. Size selection was performed with Blue Pippin (Sage Science) to remove fragments shorter than 10 kb using a 0.75% agarose cassette, the S1 marker and a high-pass protocol (Biozym, 342BLF7510). A further clean-up with AMPure XP beads (Beckman Coulter) on the size-selected DNA was performed at a 1× ratio for one library. The fragment size was assessed with the Genomic DNA 165 kb Analysis Kit on a FemtoPulse (Agilent) and the concentration of DNA was assessed using the dsDNA HS assay on a Qubit 3 fluorometer (Thermo Fisher). For each of the three sequencing runs, one library was prepared with the SQK-LSK109 Ligation Sequencing kit (ONT) per flow cell following the instructions of the ‘1D genomic DNA by ligation’ protocol from ONT. Briefly, 1.1 to 1.3 μg genomic DNA was used for the DNA repair reaction with the NEBNext Ultra II End Repair/dA-Tailing Module (New England Biolabs, E7546S) and the NEBNext FFPE DNA Repair Module (NEB, M6630S). On clean-up with AMPure XP beads (Beckman Coulter) at 1× ratio, the end-repaired DNA was incubated for 1 h at room temperature with Adapter Mix (ONT, SQK-LSK109), Ligation Buffer (ONT, SQK-LSK109) and the NEBNext Quick Ligation Module (NEB, E6056S). The ligation reaction was purified with AMPure XP beads (Beckman Coulter) at a 0.4× ratio and L Fragment Buffer (ONT, SQK-LSK109). A 600 ng (25 fmol) quantity of each generated library was loaded into the flow cell (FLO-PR002) on a PromethION instrument (ONT) following the manufacturer’s instructions. The Nanopore reads were base-called using GUPPY (v.3.0.3) with the high accuracy FLIP-FLOP model.

### Reporting Summary

Further information on research design is available in the Nature Research Reporting Summary linked to this article.

## Online content

Any methods, additional references, Nature Research reporting summaries, source data, extended data, supplementary information, acknowledgements, peer review information; details of author contributions and competing interests; and statements of data and code availability are available at 10.1038/s41587-020-00747-w.

### Supplementary information

Supplementary Information Supplementary Figs. 1–13, Tables 1–17 and Sections 1 and 2.

Reporting Summary

### Source data

Source Data Fig. 2 Tabulated data.

Source Data Fig. 3 Tabulated data.

Source Data Fig. 4 Tabulated data.

## Data Availability

All sequence datasets and de novo genome assemblies described in the manuscript are publicly available through the corresponding repositories. Specific hyperlinks for the four human datasets are provided in the Supplementary Information: Supplementary Table 1 provides hyperlinks for all of the long-read datasets; Supplementary Table 2 provides hyperlinks for all of the short-read datasets; Supplementary Table 3 provides hyperlinks for all of the de novo assemblies used in the benchmark; Supplementary Table 16 provides hyperlinks for the BAC/fosmid sequences used for consensus quality assessment. The BIONANO data of CHM13 are available at https://github.com/nanopore-wgs-consortium/CHM13. Specific hyperlinks for the non-human datasets are provided in Supplementary Table 17. The supplementary files, including all of the WENGAN assemblies described in the manuscript, are available through Zenodo at https://zenodo.org/record/3779515. The specific commands for each WENGAN assembly are provided in Supplementary Subsection 1.2. The NovaSeq 6000, MGISEQ-2000RS and PromethION sequence data of NA12878 were submitted to the Sequence Read Archive under the BioProject accession number PRJNA603060. Source data are provided with this paper.

## Code availability

The WENGAN code (v.0.2) used in this manuscript is freely available at https://github.com/adigenova/wengan and is distributed under the MIT open source license, and at CODE OCEAN (10.24433/CO.9469612.v1).
