# Supplementary material for: Efficient hybrid de novo assembly of human genomes with WENGAN
Source: Nat Biotechnol. 2020 Dec 14;39(4):422–30. doi: 10.1038/s41587-020-00747-w (PMC8041623; doi:10.1038/s41587-020-00747-w)
Supplement: Supplementary file 1 — Supplementary Figs. 1–13, Tables 1–17 and Sections 1 and 2. [file 41587_2020_747_MOESM1_ESM.pdf]

nature research

https://doi.org/10.1038/s41587-020-00747-w

Supplementary information

Efficient hybrid de novo assembly of human genomes with WENGAN

In the format provided by the authors and unedited

Supplementary Material: ”Efficient hybrid de novo assembly of human genomes with WENGAN”

Alex Di Genova[1,2], Elena Buena-Atienza[3,4], Stephan Ossowski[3,4] and Marie-France Sagot[1,2]

[1]*Inria Grenoble Rhoñe-Alpes, 655, Avenue de l'Europe, 38334 Montbonnot, France.*

[2]*Université de Lyon, Université Lyon 1, CNRS, Laboratoire de Biométrie et Biologie Evolutive UMR 5558, F-69622 Villeurbanne, France*

[3]*Institute of Medical Genetics and Applied Genomics, University of Tübingen, Tübingen, Germany.*

[4]*NGS Competence Center Tübingen (NCCT), University of Tübingen, Tübingen, Germany.*

Correspondence should be addressed to Alex Di Genova (email: digenova@gmail.com)

Contents

1 Genomes assemblies **4**

1.1 Short-read assemblies . . . 4

1.1.1 ABYSS . . . 4

1.1.2 DISCOVARDENOVO . . . 4

1.1.3 MINIA3 . . . 4

1.2 WENGAN assemblies . . . 5

1.2.1 WENGAN assemblies of NA12878, NA24385, HG00733, and CHM13 . . . 5

1.2.2 WENGAN assemblies of NA12878 at different long-read coverage . . . 6

1.2.3 WENGAN assemblies of non-human genomes . . . 7

1.3 FLYE assemblies . . . 8

1.3.1 FLYE assemblies at different long-read coverage . . . 8

2 Polishing FLYE assemblies **8**

2.1 Polishing with short and long reads . . . 8

List of Figures

Supplementary Figure 1 Example of a chimeric contig detected by INTERVALMISS . 24
Supplementary Figure 2 Chimeric MINIA3 contigs detected by INTERVALMISS . . . 25
Supplementary Figure 3 Spectrum of synthetic mate-pair libraries 26
Supplementary Figure 4 Concordance of the mate-edge lengths before and after gap filling 27
Supplementary Figure 5 NGx and NGAx of CHM13 assemblies 27
Supplementary Figure 6 Analysis of Segmental Duplication sequences resolved in the CHM13 assemblies 28
Supplementary Figure 7 DotPlots of contigs mapped to the CHM13 T2T-X chromosome (v.07) 29
Supplementary Figure 8 The MHC Region is spanned in a single contig in the CHM13 assemblies 30
Supplementary Figure 9 NGx and NGAx of NA12878, HG00733, and NA24385 assemblies 31
Supplementary Figure 10 Distribution of the WENGAN consensus errors 32
Supplementary Figure 11 Assembly of the complex MHC region in diploid genomes . 33
Supplementary Figure 12 Analysis of Segmental Duplication sequences resolved in diploid genomes 34
Supplementary Figure 13 Assembly of the complex MHC region at different long-read coverage 35

List of Tables

Supplementary Table 1 Long read data 10
Supplementary Table 2 Short read data 11
Supplementary Table 3 Public long-read and hybrid assemblies used for benchmarking 12
Supplementary Table 4 QUAST validation of the CHM13 assemblies 13
Supplementary Table 5 BAC evaluation of the CHM13 assemblies 14
Supplementary Table 6 Repeat class benchmark using the CHM13 T2T-X chromosome (v.07) 14
Supplementary Table 7 Short read assemblies 15
Supplementary Table 8 QUAST validation of the diploid assemblies 16
Supplementary Table 9 BAC/Fosmid evaluation of the NA12878 (rel5) and HG00733 assemblies 17
Supplementary Table 10 Polishing the FLYE(v2.4) assembly of NA12878 with short (HiSeq 50X) and long (ONT rel5 40X) reads 18
Supplementary Table 11 Long-read coverage tritiation 18
Supplementary Table 12 WENGAN and FLYE assemblies of NA12878 at different long-read coverage. 19
Supplementary Table 13 Fosmid evaluation of the WENGAN and FLYE assemblies of NA12878 (flipflop dataset) 20
Supplementary Table 14 Polishing the FLYE(v2.5) assembly of NA12878 with short (NovaSeq 53X) and long (ONT flipflop 30X) reads 21
Supplementary Table 15 WENGAN assemblies of non-human genomes 22
Supplementary Table 16 BAC and Fosmid sequences used to assess consensus accuracy 22
Supplementary Table 17 Short and long reads datasets of non-human genomes 23

1 Genomes assemblies

1.1 Short-read assemblies

1.1.1 ABYSS

```
#Abyss version 2.1.5

#NA12878 2x150bp HiSeq 2500
abyss-pe name=NA12878-abyss-ILL60X150 np=20 k=96 lib="pea peb" pea="BH88WKADXX.R1.fastq.gz BH88WKADXX.R2.fastq.gz"
    peb="AH81VLADXX.R1.fastq.gz AH81VLADXX.R2.fastq.gz" B=40G H=4 kc=3 v=-v contigs
#NA12878 2x150bp NovaSeq
abyss-pe name=NA12878-abyss-NS np=20 k=96 lib="pea" pea="S22_L001_R1_001.fastq.gz S22_L001_R2_001.fastq.gz" B=40G H=4 kc=3 v=-v
    contigs
# NA12878 2x150bp MGI-2000
abyss-pe name=NA12878-abyss-MGI np=20 k=96 lib="pe1 pe2" pe1="NA12878EBA.bgi.fwd.fastq.gz NA12878EBA.bgi.rev.fastq.gz"
    pe2="MGISEQ.sample.fwd.gz MGISEQ.sample.rev.gz" B=40G H=4 kc=3 v=-v contigs
```

1.1.2 DISCOVARDENOVO

```
#Discovar version discovarexp-51885
DISCO=/path/DiscovarExp
export MALLOC_PER_THREAD=1
#NA12878 2x250bp HiSeq 2500
${DISCO} READS="SRR891258_{1,2}.fastq.gz,SRR891259_{1,2}.fastq.gz" NUM_THREADS=44 OUT_DIR=60XNA12878
#NA12878 2x150bp NovaSeq
 ${DISCO} READS="S22_L001_R{1,2}_001.fastq.gz" NUM_THREADS=44 OUT_DIR=NA12878NS
# NA12878 2x150bp MGI-2000
${DISCO} READS="NA12878EBA.bgi.R{1,2}.fastq.gz,MGISEQ.sample.R{1,2}.fastq.gz" NUM_THREADS=44 OUT_DIR=NA12878MGI
# NA24385
# prior to assembly the reads shorter than 100 bp were discarded with fastp
fastp -l 100 -i D1_S1S2_R1.fastq.gz -I D1_S1S2_R2.fastq.gz -o D1_S1S2T_R1.fastq.gz -O D1_S1S2T_R2.fastq.gz
${DISCO} READS="D1_S1S2T_R{1,2}.fastq.gz" NUM_THREADS=44 OUT_DIR=60XNA24385
# HG00733
${DISCO} READS="SRR5534476_{1,2}.fastq.gz,SRR5534475_{1,2}.fastq.gz" NUM_THREADS=44 OUT_DIR=60XHG00733
#CHM13
${DISCO} READS="SRR3189741_{1,2}.fastq.gz,SRR3189742_{1,2}.fastq.gz" NUM_THREADS=44 OUT_DIR=60XCHM13
```

1.1.3 MINIA3

```
# Minia 3, git commit 017d23e
#NA12878 2x150bp HiSeq 2500
echo BH88WKADXX.R1.fastq.gz BH88WKADXX.R2.fastq.gz AH81VLADXX.R1.fastq.gz AH81VLADXX.R2.fastq.gz | xargs -n 1 > reads.txt
# the script run-minia.pl runs a mult-K assembly with kmer-sizes 41,81,121 and min k-mer frequencies of 2,2,2 respectively.
perl run-minia.pl -a reads.txt -c 20 -p NA12878-Hiseq
#NA12878 2x150bp NovaSeq
```

```
echo S22_L001_R1_001.fastq.gz S22_L001_R2_001.fastq.gz | xargs -n 1 > reads.txt
perl run-minia.pl -a reads.txt -c 20 -p NA12878-NS
# NA12878 2x150bp MGI-2000
echo NA12878EBA.bgi.fwd.fastq.gz NA12878EBA.bgi.rev.fastq.gz MGISEQ.sample.fwd.gz MGISEQ.sample.rev.gz | xargs -n 1 > reads.txt
perl run-minia.pl -a reads.txt -c 20 -p NA12878-MGI
```

The script *run-minia.pl* is part of the WENGAN code (directory aux_scripts/run-minia.pl).

1.2 WENGAN assemblies

1.2.1 WENGAN assemblies of NA12878, NA24385, HG00733, and CHM13

```
#NA12878
#WenganA
wengan.pl -x ontlon -a A -s AH81VLADXX.R1.fastq.gz,AH81VLADXX.R2.fastq.gz,BH88WKADXX.R1.fastq.gz,BH88WKADXX.R2.fastq.gz -l
      na12878.rel5.fastq.gz -p na12878Wa -t 20 -g 3000
#WenganD high memory machine
wengan.pl -x ontlon -M 5000 -a D -s SRR891259_1.fastq.gz,SRR891259_2.fastq.gz,SRR891258_1.fastq.gz,SRR891258_2.fastq.gz -l
      na12878.rel5.fastq.gz -p na12878Wd -t 44 -g 3000
#WenganM
perl wengan.pl -x ontlon -a M -s AH81VLADXX.R1.fastq.gz,AH81VLADXX.R2.fastq.gz,BH88WKADXX.R1.fastq.gz,BH88WKADXX.R2.fastq.gz -l
      na12878.rel5.fastq.gz -p na12878Wm -t 20 -g 3000

#NA24385
#upto 500kb
LIBS=500,1000,2000,3000,4000,5000,6000,7000,8000,10000,15000,20000,30000,40000,50000,60000,70000,80000,90000,100000,120000,150000,
      180000,200000,250000,300000,350000,400000,450000,500000
#run the WenganD pipeline from the given Discovar contigs
perl wengan.pl -x ontlon -M 5000 -P 100000 -a D -s D1_S1S2T_R1.fastq.gz,D1_S1S2T_R2.fastq.gz -l ultra-long-ont.fastq.gz -p
      NA24385Wd -t 20 -g 3000 -c 60XNA24385.disco.fa -i ${LIBS}

#HG00733
#upto 80Kb
LIBS=500,1000,2000,3000,4000,5000,6000,7000,8000,10000,15000,20000,30000,40000,50000,60000,70000,80000
#run the WenganD pipeline from the given Discovar contigs
perl wengan.pl -x pacraw -a D -M 5000 -s SRR5534475_1.fastq.gz,SRR5534475_2.fastq.gz,SRR5534476_1.fastq.gz,SRR5534476_2.fastq.gz
      -l SRR7615963_subreads.fastq.gz -p HG00733Wd -t 20 -g 3000 -c HG00733.ILL250.DISCOVAR.fa -i ${LIBS}

#CHM13
#upto 500kb
LIBS=500,1000,2000,3000,4000,5000,6000,7000,8000,10000,15000,20000,30000,40000,50000,60000,70000,80000,90000,100000,120000,150000,
      180000,200000,250000,300000,350000,400000,450000,500000
#run the WenganD pipeline from the given Discovar contigs
perl wengan.pl -x ontlon -M 10000 -U 1.75 -R 1.25 -a D -s
      SRR3189741_1.fastq.gz,SRR3189741_2.fastq.gz,SRR3189742_1.fastq.gz,SRR3189742_2.fastq.gz -l ont.longest50X.rel3.fastq.gz -p
      CHM13Wd -t 44 -g 3000 -c 60XCHM13L.disco.fa -i ${LIBS}

# Hifi + Ultra-long reads (rel3)
HIFI_LIBS=500,1000,2000,3000,4000,5000,6000,7000,8000,10000,12000,15000,18000
#ultra-long libs
LIBS=500,1000,2000,3000,4000,5000,6000,7000,8000,10000,15000,20000,30000,40000,50000,60000,70000,80000,90000,100000,120000,150000,
      180000,200000,250000,300000,350000,400000,450000,500000
perl wengan.pl -x ccsont -b HIFI-20kb.fastq.gz -a M -l rel3.longest_50X.fastq.gz -p CHM13.WenganM.Hifi_UL -g 3000 -t 44 -I
      ${HIFI_LIBS} -i ${LIBS}
```

1.2.2 WENGAN assemblies of NA12878 at different long-read coverage

The command used for the WENGAN assemblies with MGI+ONT data are shown. Identical commands were used for the WENGAN assemblies from ILL+ONT data.

```
# WenganA
#10X
perl wengan.pl -M 1000 -N 2 -x ontraw -a A -s short.reads -l 10X.fastq.gz -p 10X -g 3000 -t 20 2>10X.err > 10X.log
#15X
perl wengan.pl -M 1000 -N 3 -x ontraw -a A -s short.reads -l 15X.fastq.gz -p 15X -g 3000 -t 20 2>15X.err > 15X.log
#20X
perl wengan.pl -M 1000 -N 4 -x ontraw -a A -s short.reads -l 20X.fastq.gz -p 20X -g 3000 -t 20 2>20X.err > 20X.log
#25X
perl wengan.pl -M 1000 -N 5 -x ontraw -a A -s short.reads -l 25X.fastq.gz -p 25X -g 3000 -t 20 2>25X.err > 25X.log
#30X
perl wengan.pl -M 1000 -N 5 -x ontraw -a A -s short.reads -l 30X.fastq.gz -p 30X -g 3000 -t 20 2>30X.err > 30X.log

# WenganD
#10X
perl wengan.pl -N 2 -x ontraw -a D -s short.reads -l 10X.fastq.gz -p 10X -g 3000 -t 44 2>10X.err > 10X.log
#15X
perl wengan.pl -N 3 -x ontraw -a D -s short.reads -l 15X.fastq.gz -p 15X -g 3000 -t 44 2>15X.err > 15X.log
#20X
perl wengan.pl -N 4 -x ontraw -a D -s short.reads -l 20X.fastq.gz -p 20X -g 3000 -t 44 2>20X.err > 20X.log
#25X
perl wengan.pl -N 5 -x ontraw -a D -s short.reads -l 25X.fastq.gz -p 25X -g 3000 -t 44 2>25X.err > 25X.log
#30X
perl wengan.pl -N 5 -x ontraw -a D -s short.reads -l 30X.fastq.gz -p 30X -g 3000 -t 44 2>30X.err > 30X.log

# WenganM
#10X
perl wengan.pl -M 1000 -N 2 -x ontraw -a M -s short.reads -l 10X.fastq.gz -p 10X -g 3000 -t 20 2>10X.err > 10X.log
#15X
perl wengan.pl -M 1000 -N 3 -x ontraw -a M -s short.reads -l 15X.fastq.gz -p 15X -g 3000 -t 20 2>15X.err > 15X.log
#20X
perl wengan.pl -M 1000 -N 4 -x ontraw -a M -s short.reads -l 20X.fastq.gz -p 20X -g 3000 -t 20 2>20X.err > 20X.log
#25X
perl wengan.pl -M 1000 -N 5 -x ontraw -a M -s short.reads -l 25X.fastq.gz -p 25X -g 3000 -t 20 2>25X.err > 25X.log
#30X
perl wengan.pl -M 1000 -N 5 -x ontraw -a M -s short.reads -l 30X.fastq.gz -p 30X -g 3000 -t 20 2>30X.err > 30X.log
```

1.2.3 WENGAN assemblies of non-human genomes

The command used for the WENGAN assemblies of non-human genomes are shown. All non-human genomes were performed using 20 CPUs.

```
# Arabidopsis dataset
#illumina + ONT (MinION)
# WenganM, WenganA, WenganD
 perl wengan.pl -x ontraw -a M -s short.reads -l ont.reads -p ara_Wm_or1 -t 20 -g 120
 perl wengan.pl -x ontraw -a A -s short.reads -l ont.reads -p ara_Wa_or1 -t 20 -g 120
 perl wengan.pl -x ontraw -a D -s short.reads-l ont.reads -p ara_Wd_or1 -t 20 -g 120
# Assembling Illumina + Sequel (PACBIO) read
INS=500,1000,2000,3000,4000,5000,6000,7000,8000,10000,15000,20000,25000,30000
# WenganM, WenganA, WenganD
perl wengan.pl -x pacraw -i ${INS} -a M -s short.reads -l sequel.reads -p ara_Wm_pr1 -t 20 -g 120
perl wengan.pl -x pacraw -i ${INS} -a A -s short.reads -l sequel.reads -p ara_Wa_pr1 -t 20 -g 120
perl wengan.pl -x pacraw -i ${INS} -a D -s short.reads -l sequel.reads -p ara_Wd_pr1 -t 20 -g 120

# Drosophila dataset
# illumina + ONT (MinION)
# WenganM, WenganA, WenganD
perl wengan.pl -x ontraw -a M -M 1000 -d 3 -f 0.5 -s short.reads -l ont.reads -p dro_Wm_or1 -t 20 -g 150
perl wengan.pl -x ontraw -a A -M 1000 -d 3 -s short.reads -l ont.reads -p dro_Wa_or1 -t 20 -g 150
perl wengan.pl -x ontraw -a D -s short.reads -l ont.reads -p dro_Wd_or1 -t 20 -g 150

# Fish dataset
# illumina + ONT (MinION)
# WenganM, WenganA, WenganD
perl wengan.pl -x ontraw -d 4 -M 1000 -T 10000 -a M -s short.reads -l ont.reads -p fish_Wm_or1 -t 20 -g 500
perl wengan.pl -x ontraw -d 4 -a A -s short.reads -l ont.reads -p fish_Wa_or1 -t 20 -g 500
perl wengan.pl -x ontraw -a D -s short.reads -l ont.reads -p fish_Wd_or1 -t 20 -g 500
```

1.3 Flye assemblies

1.3.1 Flye assemblies at different long-read coverage

```
# Flye v2.5
#10X
flye --nano-raw 10X.fastq.gz -o 10X.flye -t 44 -g 3g
#15X
flye --nano-raw 15X.fastq.gz -o 15X.flye -t 44 -g 3g
#20X
flye --nano-raw 20X.fastq.gz -o 20X.flye -t 44 -g 3g
#25X
flye --nano-raw 25X.fastq.gz -o 25X.flye -t 44 -g 3g
#30X
flye --nano-raw 30X.fastq.gz -o 30X.flye -t 44 -g 3g
```

2 Polishing Flye assemblies

2.1 Polishing with short and long reads

The Flye assemblies of NA12878 were polished using Racon and ntEdit. In particular, two rounds of long-read polishing with Racon were performed, followed by three rounds of short-read polishing with ntEdit. The commands executed were the following:

```
#Polishing of the Flye assembly with 40X Nanopore reads (rel5) and 50X of short illumina reads.
make -f polish.mk PREF=na12878.flye.racon ASM=FLYE.NA12878.fa CPU=44 READS=na12878.rel5.fastq.gz SREADS="AH81VLADXX.R1.fastq.gz
      AH81VLADXX.R2.fastq.gz BH88WKADXX.R1.fastq.gz BH88WKADXX.R2.fastq.gz" all
#Polishing of the Flye assembly at 30X coverage with flip-flop called Nanopore reads and Illumina Novaseq short-reads
make -f polish.mk PREF=na12878.flye30x.racon ASM=na12878.flye30X.fa CPU=44 READS=30X.fastq.gz SREADS="S22_L001_R1_001.fastq.gz
      S22_L001_R2_001.fastq.gz" all
```

The makefile *polish.mk* contains the following instructions:

```
.DELETE_ON_ERROR:
#racon version v1.4.9
RACON=/path/racon
#minimap2 version 2.15-r905
MM=/path/minimap2
# nthits version 0.1.0
NTH=/path/nthits
# ntedit version 1.2.3
NTE=/path/ntedit
TIME=/path/time
#LONG-READ POLISHING
#first round of long-read polishing
$(PREF).r1.paf:
	${TIME} -v -o $(PREF).r1.mm.time ${MM} -x map-ont -t ${CPU} ${ASM} ${READS} > $@
$(PREF).r1.fa:$(PREF).r1.paf
	${TIME} -v -o $(PREF).r1.racon.time ${RACON} -u -t ${CPU} ${READS} $< ${ASM} > $@ 2> $(PREF).r1.racon.log
#second round of long-read polishing
$(PREF).r2.paf:$(PREF).r1.fa
	${TIME} -v -o $(PREF).r2.mm.time ${MM} -x map-ont -t ${CPU} $< ${READS} > $@
$(PREF).r2.fa:$(PREF).r2.paf
	${TIME} -v -o $(PREF).r2.racon.time ${RACON} -t ${CPU} ${READS} $< $(PREF).r1.fa > $@ 2> $(PREF).r2.racon.log
#SHORT-READ POLISHING
$(PREF).nthits_k60.bf:$(PREF).r2.fa
	echo ${SREADS} | xargs -n 1 > shortreads.txt
	${TIME} -v -o $(PREF).nthits.K40.time ${NTH} -b 36 -k 40 -t ${CPU} -p $(PREF).nthits --outbloom --solid @shortreads.txt
	${TIME} -v -o $(PREF).nthits.K50.time ${NTH} -b 36 -k 50 -t ${CPU} -p $(PREF).nthits --outbloom --solid @shortreads.txt
	${TIME} -v -o $(PREF).nthits.K60.time ${NTH} -b 36 -k 60 -t ${CPU} -p $(PREF).nthits --outbloom --solid @shortreads.txt
# three rounds of short-read polishing with ntEdit after two round of RACON
$(PREF).r2.nt3_edited.fa:$(PREF).r2.fa $(PREF).nthits_k60.bf
	${TIME} -v -o $(PREF).r2.nt1.time ${NTE} -t ${CPU} -k 60 -i 5 -d 5 -b $(PREF).r2.nt1 -r $(PREF).nthits_k60.bf -f $<
	${TIME} -v -o $(PREF).r2.nt2.time ${NTE} -t ${CPU} -k 50 -i 5 -d 5 -b $(PREF).r2.nt2 -r $(PREF).nthits_k50.bf -f
		$(PREF).r2.nt1_edited.fa
	${TIME} -v -o $(PREF).r2.nt3.time ${NTE} -t ${CPU} -k 40 -i 5 -d 5 -b $(PREF).r2.nt3 -r $(PREF).nthits_k40.bf -f
		$(PREF).r2.nt2_edited.fa
# all done
all: $(PREF).r2.nt3_edited.fa
```

Supplementary Table 1: Long read datasets used to evaluate the performance of WENGAN. The ultra-long CHM13 dataset (Rel3) was subsampled to the longest reads covering 50X of the human genome.

	NA12878		CHM13		HG00733	NA24385
Technology	ONT		ONT	PacBio/HiFi	PacBio	ONT
Machine	PromethION	MinION	MinION	Sequel II	Sequel	MinION
Read count	10,446,475	11,628,512	1,253,933	5,566,855	12,554,013	7,762,763
Min read Size	2,000	2,000	66,232	2,000	2,000	2,000
Max read Size	3,323,027	1,019,957	6,598,569	50,003	158,025	2,151,856
N50	17,181	13,643	123,269	17,781	33,201	54,069
N75	9,645	8,385	88,390	15,978	20,888	24,390
Coverage	35	40	50	33	90	60
# reads $>$ 100kb	1,618	56,283	606,249	0	1,176	310,435
Coverage reads $>$ 100kb	0.10	3.29	32.06	0	0.04	18.08
Source	PRJNA603060	ONT Rel5	T2T	SRR112921[20-23]	SRR7615963	GIAB
URL	PRJNA603060	M, P, U	Rel3	SRA	ENA	GIAB:final

Supplementary Table 2: Short read datasets used to evaluate the performance of WENGAN.

Sample	Technology	Machine	Read count	File	Read length	Coverage	Source/URL
NA12878	Illumina	HiSeq 2000	186,421,465	SRR891258_1.fastq.gz	250	59.97	PRJNA196624
			186,421,465	SRR891258_2.fastq.gz			
			185,398,624	SRR891259_1.fastq.gz			
			185,398,624	SRR891259_2.fastq.gz			
	Illumina	HiSeq 2500	266,077,618	AH81VLADXX.R1.fastq.gz	150	50.22	GIAB
			266,077,618	AH81VLADXX.R2.fastq.gz			
			252,850,306	BH88WKADXX.R1.fastq.gz			
			252,850,306	BH88WKADXX.R2.fastq.gz			
	Illumina	NovaSeq	548,283,470	S22_L001_R1_001.fastq.gz	150	53.06	PRJNA603060
			548,283,470	S22_L001_R1_001.fastq.gz			
	MGI	MGISEQ-2000	376,183,716	EBA.bgi.fwd.fastq.gz	150	36.40	PRJNA603060
			376,183,716	EBA.bgi.rev.fastq.gz			
			172,099,754	V100003043_L01_1.fq.gz	150	16.65	GIAB
			172,099,754	V100003043_L01_2.fq.gz			
NA24385	Illumina	HiSeq 2500	441,957,241	D1_S1S2_R1.fastq.gz	250	71.28	GIAB
			441,957,241	D1_S1S2_R2.fastq.gz			
CHM13	Illumina	HiSeq 2500	202,861,861	SRR3189742_1.fastq.gz	250	66.11	PRJNA269593
			202,861,861	SRR3189742_2.fastq.gz			
			206,992,396	SRR3189741_1.fastq.gz			
			206,992,396	SRR3189741_2.fastq.gz			
HG00733	Illumina	HiSeq 2500	196,489,884	SRR5534476_1.fastq.gz	250	63.26	PRJNA300840
			196,489,884	SRR5534476_2.fastq.gz			
			195,745,350	SRR5534475_1.fastq.gz			
			195,745,350	SRR5534475_2.fastq.gz			
Total	-		6,462,723,370	-	-	416.96	-

Supplementary Table 3: Public long-read and hybrid assemblies of NA12878 (rel5), HG00733 (sequel and ONT), NA24385 (ONT), and CHM13 (ONT and HiFi) used for benchmarking. All the assemblies were done by the assembler developers.

Sample	Assembler	Version	URL	Accessed
NA12878	WTDBG2	2.3	NA12878.wt.fa.gz	27/02/2019
	MASURCA	3.2.8	MaSuRCA_3.2.8_nanopore.rel5.fa	27/02/2019
	FLYE	2.4	na12878.ont-ul.35x.fasta.gz	27/03/2019
	CANU	1.7	albacore_canu_nanopolish2_pilon2_racon2.fasta	27/02/2019
HG00733	FALCON	Unzip v. July-2018	RBJD01.fasta.gz	03/05/2019
	SHASTA	0.1	HG00733_shasta_marginpolish_helen.fa	25/03/2020
NA243875	SHASTA	0.1	HG002_shasta_marginpolish_helen.fa	25/03/2020
CHM13	SHASTA (ONT-REL3)	0.1	shasta.contigs.rel3.fasta.gz	25/03/2020
	CANU (CURATED)	1.7.1	chm13.draft_v0.7.fasta.gz	25/03/2020
	CANU (ONT-REL3)	1.9	canu.contigs.rel3.fasta.gz	25/03/2020
	FLYE (ONT-REL3)	2.5	flye.contigs.rel3.fasta.gz	25/03/2020
	CANU (HIFI-20KB)	hicanu_rc	chm13_20k_canu_hifi.fasta.gz	25/03/2020
	PEREGRINE (HIFI-20KB)	0.1.5.3	chm13_20k_peregrine_hifi.fasta.gz	25/03/2020
	HICANU (HIFI-20KB)	hicanu_rc	chm13_20k_hicanu_hifi.fasta.gz	25/03/2020

Supplementary Table 4: QUAST validation of the CHM13 assemblies. NG50 is the contig length such that using longer contigs produces half (50%) of the bases of the reference GRCh38 (3.0882 Gb) genome. NGA50 is NG50 where the lengths of aligned blocks are counted instead of the contig lengths. LG50 is the minimum number of contigs that produce half of the reference length. LGA50 is similar to LG50 but aligned blocks are counted instead. Assembly-errors correspond to the number of positions in the assembled contigs where the left flanking sequence aligns over 1 kbp away from the right flanking sequence on the reference (relocation), or they overlap on more than 1 kbp (relocation), or else the flanking sequences align on different strands (inversion) or different chromosomes (translocation). Genome fraction (%) is the total number of bases aligned of the reference, divided by the reference size. The QUAST (Version: 5.0.2) analysis was run with the options min-identity 80 and fragmented using the autosomes plus X and Y chromosomes of GRCh38 ("quast -r GRCh38_chrom_no_alt.fa –large –min-identity 80 –fragmented"). Additionally, we ran a QUAST analysis using as reference the curated CHM13 Canu assembly (chm13.draft_v0.7, 2.9384 Gb) generated by the T2T consortium. Assembly errors overlapping centromeres or segmental duplications of GRCh38 were discounted. A second QUAST run using a minimum alignment length of 50kb was performed to discount assembly errors overlapping problematic regions (segmental duplications and centromeres) of the curated CHM13 assembly. Assembly errors before and after discounting problematic regions are shown.

Assembler	NG50 (Mb)	LG50	Assembly errors	Unaligned length (Mb)	Genome fraction (%)	Duplication ratio	Indels per 100kb	Largest alignment (Mb)	NGA50 (Mb)	LGA50	CPU Hours	Max RAM (Gb)	Elapsed time (h:m:s)
QUAST Reference	GRCh38 (Length 3,088,269,832 bp)												
CANU (UL)	74.06	16	8,157 / 153	64.36	96.804	1.029	322.97	70.9	25.37	38	∼219,000	80	-
FLYE (UL)	69.64	17	4,837 / 106	37.85	96.373	1.018	451.33	90.7	26.21	37	∼5,000	∼871	-
SHASTA (UL)	47.76	19	649 / 78	1.82	95.694	1.004	150.9	90.5	25.87	37	-	∼2,000	∼24:00:00
CANU (HiFi)	45.63	20	11,680 / 171	74.74	97.431	1.051	33.79	71.8	22.39	41	3,524	80	∼12:00:00
HICANU (HiFi)	77.12	14	12,401 / 193	83.18	97.446	1.120	39.66	90.5	25.06	37	5,000	119	∼12:00:00
PEREGRINE (HiFi)	37.30	26	3,209 / 149	23.02	96.205	1.018	32.72	86.7	23.85	40	58	449	2:00:00
WENGAN (ILL+UL)	69.72	16	1,117 / 105	9.79	95.634	1.008	35.29	90.6	23.84	41	1,198	646	38:12:30
WENGAN (HiFi+UL)	70.73	16	1,239 / 110	12.90	95.662	1.009	34.03	71.81	26.84	37	981	125	85:03:47
QUAST Reference	chm13.draft_v0.7 (Length 2,938,464,690 bp)												
CANU (UL)	77.96	15	6,136 / 373	31.40	98.392	1.027	325.97	104.4	47.44	21	∼219,000	∼80	-
FLYE (UL)	70.32	16	2,139 / 334	18.56	97.368	1.017	446	111.8	46.55	20	∼5,000	∼871	-
SHASTA (UL)	58.09	18	187 / 60	0.39	96.149	1.002	141.25	111.7	44.54	20	-	∼2,000	∼24:00:00
CANU (HiFi)	46.82	19	5,300 / 652	32.86	98.703	1.054	26.34	111.7	34.68	25	3,524	80	∼12:00:00
HICANU (HiFi)	82.40	13	5,773 / 748	38.17	98.741	1.123	32.39	111.6	39.12	22	5,000	119	12:00:00
PEREGRINE (HiFi)	38.11	24	1,194 / 188	6.80	97.261	1.014	15.76	109.9	31.44	27	58	449	2:00:00
WENGAN (ILL+UL)	71.25	15	431 / 147	3.58	96.321	1.005	16.86	110.6	35.01	24	1,198	646	38:12:30
WENGAN (HiFi+UL)	80.63	15	539 / 140	5.62	96.413	1.006	15.21	111.5	46.73	20	981	125	85:03:47

Supplementary Table 5: BAC evaluation using a total of 341 BACs (51,5Mb) of CHM13. ”Closed” refers to the number of BACs for which 99.5% of their length aligns to a single locus. The identity and phred-Quality Value (QV) metrics are computed from closed BACs only. The unique closed BACs are BACs located in unique regions of the human genome (30 BACs).

	Closed BACs		BAC bases		Closed BACs				Unique BACs (30)			
					Median Quality		Mean Quality		Median Quality		Mean Quality	
	#	%	length	%	%Identity	QV	%Identity	QV	%Identity	QV	%Identity	QV
Shasta (UL)	176	51.61	26,926,379	52.25	99.74	25.91	99.65	24.59	99.83	27.78	99.80	27.09
Flye (UL)	253	74.19	37,970,519	73.68	99.03	20.11	98.95	19.79	99.37	21.97	99.27	21.37
Canu (UL)	314	92.08	47,415,786	92.01	99.53	23.32	99.45	22.60	99.61	24.11	99.59	23.83
Peregrine (HiFi)	136	39.88	20,114,050	39.03	99.98	37.32	99.74	25.86	100.00	44.75	99.98	37.14
Canu (HiFi)	308	90.32	46,430,951	90.10	99.99	40.56	99.95	32.62	100.00	43.82	99.98	37.17
HiCanu (HiFi)	326	95.60	49,196,764	95.47	99.99	40.71	99.95	33.28	100.00	43.82	99.98	37.17
Wengan (ILL+UL)	175	51.32	26,197,247	50.84	99.81	27.31	99.35	21.84	99.98	36.06	99.95	33.25
Wengan (HiFi+UL)	168	49.26	25,368,837	49.22	99.89	29.84	99.52	23.19	99.99	42.88	99.97	35.87

Supplementary Table 6: Repeat class analysis of the Wengan assemblies of CHM13 using as reference the curated T2T-X chromosome. Assembled contigs were aligned to the T2T-X chromosome using Mash version 2.0 (” -r chrX.t2t.fa -f one-to-one -q asm.fa -s 10000 –pi 85”). Anchored contigs (Figure 7) were masked using RepeatMasker version 4.1.0 (”-species human -gff -xm -dir=asm.rm asm.anchored.fa”). The RepeatMasker report (*.tbl) was used to collect the amount of sequence masked by repeat classes in each assembly. The percentages are computed relative to the amount of repeat class sequences masked in the curated T2T-X chromosome (v.07).

	chrX T2T		Wengan (HiFi+UL)		Wengan (ILL+UL)		Shasta (UL)		Canu (UL)		Peregrine (HiFi)		HiCanu (HiFi)	
Contigs	1		2		4		8		3		15		9	
Total length (Mb)	154.27		150.90		150.52		148.10		150.88		141.51		152.23	
Bases masked (Mb)	102.27		99.08		98.88		97.22		99.02		92.68		101.01	
Repeat classes	#	(Mb)	%	(Mb)	%	(Mb)	%	(Mb)	%	(Mb)	%	(Mb)	%	(Mb)
SINEs	70,660	16.41	99.76	16.37	99.55	16.34	97.17	15.95	99.60	16.35	92.13	15.12	96.49	15.84
ALUs	46,503	12.44	99.72	12.41	99.47	12.37	96.67	12.03	99.52	12.38	91.05	11.33	95.44	11.87
MIRs	23,571	3.87	99.89	3.87	99.77	3.86	98.77	3.83	99.88	3.87	95.50	3.70	99.80	3.87
LINEs	63,846	54.75	99.92	54.71	99.60	54.53	98.77	54.08	99.67	54.57	94.26	51.61	99.68	54.57
LINE1	42,219	47.31	99.91	47.27	99.56	47.10	98.81	46.75	99.69	47.16	94.12	44.53	99.64	47.14
LINE2	18,782	6.37	100.01	6.37	99.81	6.36	98.38	6.27	99.54	6.34	94.84	6.04	99.93	6.37
L3/CR1	2,087	0.75	99.87	0.75	100.00	0.75	99.03	0.75	99.62	0.75	96.07	0.73	99.99	0.75
LTR elements	30,495	18.62	99.92	18.61	99.73	18.57	98.97	18.43	99.66	18.56	92.86	17.29	98.82	18.40
ERVL	6,459	3.90	100.03	3.90	99.74	3.89	99.33	3.87	99.76	3.89	92.45	3.61	99.34	3.87
ERVL-MaLRs	14,573	7.17	99.94	7.16	99.88	7.16	98.97	7.09	99.73	7.15	94.29	6.76	98.48	7.06
ERV_classI	6,998	6.27	99.85	6.26	99.62	6.24	98.77	6.19	99.54	6.24	91.96	5.76	98.77	6.19
ERV_classII	468	0.49	99.97	0.49	98.73	0.49	98.42	0.49	99.61	0.49	82.49	0.41	98.77	0.49
DNA elements	26,013	6.31	99.87	6.31	99.87	6.31	98.86	6.24	99.56	6.29	95.78	6.05	99.46	6.28
hAT-Charlie	12,551	2.58	99.88	2.58	99.78	2.58	98.73	2.55	99.69	2.58	95.85	2.48	99.47	2.57
TcMar-Tigger	6,255	2.20	100.02	2.20	100.07	2.21	99.17	2.19	99.40	2.19	95.19	2.10	99.54	2.19
Unclassified	489	0.25	100.55	0.25	99.89	0.25	97.02	0.24	98.64	0.24	91.72	0.23	99.07	0.25
Total interspersed repeats	-	96.35	99.89	96.25	99.63	95.99	98.54	94.94	99.65	96.01	93.72	90.29	98.95	95.34
Small RNA	724	0.07	100.06	0.07	99.91	0.07	97.98	0.07	101.23	0.08	95.18	0.07	99.42	0.07
**Satellites**	95	3.78	18.28	0.69	18.21	0.69	6.15	0.23	24.11	0.91	13.70	0.52	98.64	3.73
Simple repeats	29,163	1.80	100.26	1.80	102.90	1.85	95.95	1.72	96.57	1.73	87.04	1.56	90.89	1.63
Low complexity	3,718	0.26	98.32	0.25	98.64	0.25	92.50	0.24	105.59	0.27	84.22	0.22	85.51	0.22

Supplementary Table 7: Short read assemblies. The ABYSS2 and MINIA3 assemblies were run using 20 CPUs. The DISCOVAR assemblies were run using 44 CPUs and a high memory machine.

**Sample**	**Sequencer**	**Assembler**	**Contigs**			**NG50**	**NG75**	**Size**	**CPU**	**Elapsed**	**RAM**
			**Total**	**Min**	**Max**	**(bp)**	**(bp)**	**(Mb)**	**hours**	**time**	**(Gb)**
NA12878	HiSeq 2500 (2x150bp)	MINIA3	465,278	500	160,556	9,680	3,524	2,715	77	10:00:15	16
NA12878	NovaSeq (2x150bp)	MINIA3	402,298	500	189,686	11,797	4,323	2,722	69	8:03:09	15
NA12878	MGISeq (2x150bp)	MINIA3	428,404	500	192,702	10,568	3,739	2,701	73	11:01:35	21
NA12878	HiSeq 2500 (2x150bp)	ABYSS2	363,092	500	176,953	12,941	4,884	2,715	572	32:50:55	43
NA12878	NovaSeq (2x150bp)	ABYSS2	333,295	500	205,648	14,558	5,512	2,725	436	24:13:45	43
NA12878	MGISeq (2x150bp)	ABYSS2	430,581	500	192,271	10,789	3,638	2,682	437	24:12:22	43
NA12878	HiSeq 2000 (2x250bp)	DISCOVAR	145,032	500	768,671	91,438	38,345	2,863	439	14:50:28	622
NA12878	NovaSeq (2x150bp)	DISCOVAR	147,907	500	553,546	49,129	20,438	2,774	439	17:24:36	595
NA12878	MGISeq (2x150bp)	DISCOVAR	157,838	500	494,557	43,456	16,964	2,758	410	17:33:48	585
NA24385	HiSeq 2500 (2x250bp)	DISCOVAR	157,761	500	774,130	81,714	36,386	2,888	577	21:18:12	651
CHM13	HiSeq 2500 (2x250bp)	DISCOVAR	111,955	500	823,426	97,044	42,421	2,853	723	23:04:00	647
HG00733	HiSeq 2500 (2x250bp)	DISCOVAR	182,375	500	737,710	54,033	22,394	2,856	450	17:28:12	644

Supplementary Table 8: QUAST validation of the diploid assemblies. NG50 is the contig length such that using longer contigs produces half (50%) of the bases of the reference GRCh38 (3.0882 Gb) genome. NGA50 is NG50 where the lengths of aligned blocks are counted instead of the contig lengths. LG50 is the minimum number of contigs that produce half of the reference length. LGA50 is similar to LG50 but aligned blocks are counted instead. Assembly-errors correspond to the number of positions in the assembled contigs where the left flanking sequence aligns over 1 kbp away from the right flanking sequence on the reference (relocation), or they overlap on more than 1 kbp (relocation), or else the flanking sequences align on different strands (inversion) or different chromosomes (translocation). Genome fraction (%) is the total number of bases aligned of the reference, divided by the reference size. The QUAST (Version: 5.0.2) analysis was run with the options min-identity 80 and fragmented using the autosomes plus X and Y chromosomes of GRCh38 ("quast -r GRCh38_chrom_no_alt.fa –large –min-identity 80 –fragmented"). Assembly errors overlapping centromeres or segmental duplications of GRCh38 were discounted. Assembly errors before and after discounting problematic regions are shown. The SHASTA assemblies were generated and polished using only Nanopore reads. The total elapsed time for the WENGAND assemblies (using 44 cores) was HG00733(43 hours), NA34285 (43 hours), and NA12878 (23.2 hours). The total elapsed time for the WENGANA and WENGANM assemblies of NA12878 (using 20 cores) was 44.8 and 22.5 hours, respectively.

Assembler	NG50	LG50	Assembly errors	Unaligned length	Genome fraction (%)	Indels per 100kb	Largest alignment	NGA50	LGA50
NA12878 (REL5)									
WENGANA	25,991,829	31	589 / 91	4,759,777	94.30	90.36	75,324,995	14,344,805	59
WENGAND	35,310,335	26	955 / 158	8,625,359	95.25	53.48	72,841,993	16,408,877	54
WENGANM	17,237,782	44	638 / 153	5,211,387	94.22	102.36	45,660,962	11,815,416	72
MASURCA	8,425,533	105	2,622 / 275	24,677,708	95.80	47.07	32,622,531	5,692,898	149
CANU (Polished)	10,410,217	79	2,346 / 194	9,422,362	95.05	55.39	34,067,697	7,120,450	113
WTDBG2	11,842,381	62	2,074 / 124	32,133,988	91.70	1135.05	70,478,230	7,380,335	98
FLYE	22,908,596	43	3,424 / 177	21,286,450	95.56	1470.67	78,988,942	12,355,459	65
HG00733 (PACBIO)									
WENGAND	32,350,336	29	863 / 119	6,956,865	95.12	35.82	71,027,756	17,305,449	51
FALCON	22,334,437	39	2,410 / 198	15,414,934	96.06	62.19	71,678,734	14,607,765	58
SHASTA (UL-ONT)	21,707,787	40	873 / 107	6,486,246	94.98	140.97	78,219,729	12,986,946	59
HG002 (UL-ONT)									
WENGAND	50,594,311	18	1,434 / 156	10,536,361	96.36	38.78	75,562,021	24,515,399	44
SHASTA	20,346,145	36	962 / 126	6,520,569	95.61	152.17	75,647,382	14,315,298	60

Supplementary Table 9: Fosmid / BAC evaluation of the NA12878 (rel5) and HG00733 assemblies. ”Closed” refers to the number of Fosmids/BACs for which 99.5% of their length aligns to a single locus. The identity and phred-Quality Value (QV) metrics are computed from closed Fosmids/BACs only. The common closed Fosmids/BACs are the Fosmids/BACs closed by all the evaluated genome assemblies. A total of 103 Fosmids (3.92Mb) and 179 BACs (27.9Mb) were used to determine the consensus quality of NA12878 (75 common Fosmids) and HG00733 (35 common BACs) assemblies, respectively.

	Closed		bases		Closed				Common closed			
					Median Quality		Mean Quality		Median Quality		Mean Quality	
	#	%	length	%	%Identity	QV	%Identity	QV	%Identity	QV	%Identity	QV
NA12878 (Fosmids)												
WENGANA	96	93.20	3,681,736	93.73	99.83	27.74	99.47	22.77	99.86	28.41	99.67	24.79
WENGAND	96	93.20	3,677,764	93.63	99.92	30.91	99.66	24.70	99.92	31.02	99.82	27.55
WENGANM	94	91.26	3,592,857	91.47	99.80	27.07	99.42	22.35	99.84	27.84	99.54	23.38
MASURCA	100	97.09	3,819,651	97.24	99.80	26.91	99.69	25.03	99.81	27.10	99.74	25.84
CANU (Polished)	94	91.26	3,584,995	91.27	99.74	25.88	99.28	21.45	99.87	28.79	99.52	23.21
FLYE	95	92.23	3,632,548	92.48	97.73	16.43	97.61	16.21	97.71	16.41	97.62	16.24
WTDBG2	84	81.55	3,199,569	81.45	98.06	17.13	97.93	16.83	98.04	17.08	97.95	16.89
HG00733 (BACs)												
WENGAND	51	28.49	8,112,378	29.01	99.74	25.78	99.14	20.64	99.77	26.42	99.02	20.09
FALCON	80	44.69	12,313,738	44.04	99.80	26.89	99.34	21.80	99.81	27.30	99.27	21.34
SHASTA (Polished)	41	22.91	6,550,460	23.43	99.53	23.32	98.87	19.47	99.54	23.36	98.87	19.47

Supplementary Table 10: Polishing the FLYE assembly of NA12878 with short (HiSeq 2500) and long (ONT rel5) reads. The FLYE assembly was polished using two rounds of long-read polishing with RACON followed by three rounds of short-read polishing with NTEDIT. The short-read polishing was done using the same short-reads used in the WENGAN assemblies (50X of pair-end 2x150bp reads). Consensus quality statistics after each round of polishing are presented.

			FLYE	FLYE+ RACON X 1	FLYE+ RACON X 2	FLYE+ RACON X 2+ NTEDIT X 3
T. length (Mb)			2,880.84	2,847.89	2,846.09	2,850.65
Aln. length (Mb)			2,722.89	2,745.81	2,750.02	2,750.98
bases < 99% (Mb)			157.96	102.08	96.07	99.66
Indels	short [1-2]	Number	36,649,717	12,397,212	12,022,856	2,568,960
		Rate (bp)	74	221	229	1,071
	medium [3,50)	Number	2,381,191	1,279,399	1,168,244	720,564
		Rate (bp)	1,143	2,146	2,354	3,818
	large >50	Number	13,840	14,544	14,707	14,903
		Rate (bp)	196,740	188,793	186,987	184,593
Fosmid median QV			16.42	19.95	20.23	23.49
BUSCO		#Genes	2268	-	-	3680
		%Complete	55.3	-	-	89.7
Computational Resources		CPU	-	132	250	755
		RAM	-	386	386	386

Supplementary Table 11: Long-read coverage tritiation of the PromethION data of NA12878 for de novo assembly.

# reads	Min	Max	N50	N75	Bases (Mb)	Coverage
1,878,641	5,000	2,914,544	19,575	12,724	30,000	10X
2,817,267	5,000	2,914,544	19,578	12,728	45,000	15X
3,755,076	5,000	2,914,544	19,590	12,730	60,000	20X
4,694,725	5,000	3,323,027	19,590	12,729	75,000	25X
5,633,658	5,000	3,323,027	19,589	12,729	90,000	30X

Supplementary Table 12: WENGAN and FLYE (v.2.5) assemblies of NA12878 at different long-read coverage. The WENGAND and FLYE assemblies were run using 44 CPUs on a high memory machine (750Gb RAM). WENGANA and WENGANM were run using 20 CPUs. Assembly errors overlapping centromeres or segmental duplications of GRCh38 were discounted. Assembly errors before and after discounting problematic regions are shown.

Assembler	tech	depth	length (Gb)	%R.cov	NG50 (Mb)	NGA50 (Mb)	Assembly errors	indels per 100kb	indels number short (M)	indels number medium (M)	indels number large	indels rate short	indels rate medium	indels rate large (kb)	Busco % complete	CPU hours	Max RAM (Gb)	elapsed time h:m:s
WENGANA	MGI	10	2.759	93.78	2.93	2.71	601 / 117	109.83	2.285	0.496	17,677	1,199	5,524	155,048	93.59	494	43	28:44:13
WENGANA	MGI	15	2.765	93.94	8.26	6.74	629 / 95	90.32	1.943	0.426	17,421	1,416	6,459	157,867	94.23	515	43	29:53:20
WENGANA	MGI	20	2.767	94.02	11.94	8.69	641 / 95	80.34	1.728	0.396	17,328	1,593	6,959	158,863	94.47	535	43	31:07:09
WENGANA	MGI	25	2.769	94.06	14.53	10.48	660 / 108	74.69	1.603	0.379	17,398	1,719	7,276	158,369	94.66	554	43	32:09:12
WENGANA	MGI	30	2.770	94.10	15.55	10.37	679 / 108	71.56	1.530	0.370	17,477	1,802	7,452	157,737	94.62	558	43	32:22:13
WENGANA	ILL	10	2.768	93.98	3.73	3.25	653 / 104	82.28	1.633	0.436	18,294	1,681	6,306	150,109	94.52	492	43	28:21:55
WENGANA	ILL	15	2.772	94.10	10.50	7.79	674 / 100	68.93	1.405	0.390	17,999	1,961	7,068	153,004	94.79	512	43	29:31:23
WENGANA	ILL	20	2.775	94.15	14.19	10.32	682 / 106	62.2	1.266	0.370	17,947	2,179	7,447	153,666	94.74	530	43	30:46:28
WENGANA	ILL	25	2.776	94.18	16.03	11.82	670 / 107	58.5	1.184	0.360	18,018	2,330	7,671	153,098	94.71	548	43	31:41:01
WENGANA	ILL	30	2.777	94.20	16.65	11.09	687 / 108	56.29	1.134	0.354	18,023	2,434	7,802	153,157	94.76	551	43	31:51:39
WENGAND	MGI	10	2.792	94.70	6.97	5.93	759 / 114	67.82	1.352	0.349	17,619	2,046	7,919	157,012	94.88	463	585	20:56:56
WENGAND	MGI	15	2.795	94.79	15.56	11.35	811 / 113	58.54	1.199	0.315	17,480	2,313	8,818	158,665	94.98	480	585	21:55:14
WENGAND	MGI	20	2.797	94.84	16.68	12.28	822 / 120	53.27	1.086	0.298	17,439	2,555	9,310	159,113	95.00	497	585	22:52:34
WENGAND	MGI	25	2.798	94.87	17.85	12.92	823 / 123	50.22	1.020	0.291	17,432	2,722	9,539	159,227	95.13	514	585	23:46:22
WENGAND	MGI	30	2.799	94.90	18.77	12.70	794 / 126	48.74	0.985	0.288	17,455	2,822	9,649	159,191	95.05	516	585	23:46:25
WENGAND	ILL	10	2.797	94.78	8.43	6.58	770 / 143	62.58	1.233	0.334	18,550	2,246	8,281	149,287	94.86	490	595	20:40:36
WENGAND	ILL	15	2.800	94.87	16.48	12.07	798 / 123	54.22	1.094	0.305	18,482	2,537	9,086	150,167	94.96	506	595	21:34:51
WENGAND	ILL	20	2.802	94.92	18.28	13.17	837 / 129	49.6	0.999	0.292	18,474	2,783	9,515	150,425	94.88	524	595	22:35:25
WENGAND	ILL	25	2.803	94.95	19.40	13.88	897 / 119	46.83	0.936	0.285	18,431	2,967	9,760	150,759	94.83	540	595	23:27:02
WENGAND	ILL	30	2.804	94.97	21.62	14.27	903 / 118	45.51	0.904	0.281	18,546	3,076	9,888	149,913	94.76	543	595	23:31:51
WENGANM	MGI	10	2.777	94.26	2.59	2.42	726 / 140	93.97	1.946	0.445	18,123	1,415	6,191	151,937	94.40	126	37	14:22:18
WENGANM	MGI	15	2.782	94.41	7.46	6.13	717 / 115	78.24	1.662	0.386	17,765	1,662	7,156	155,537	94.93	138	37	15:06:20
WENGANM	MGI	20	2.784	94.47	10.24	7.75	768 / 131	70.1	1.489	0.360	17,723	1,858	7,677	156,058	94.91	158	37	16:19:06
WENGANM	MGI	25	2.786	94.50	11.35	8.68	779 / 137	65.72	1.390	0.348	17,658	1,992	7,961	156,804	94.79	176	38	17:23:14
WENGANM	MGI	30	2.787	94.53	12.39	9.61	795 / 132	63.22	1.331	0.340	17,602	2,082	8,136	157,368	94.93	187	44	18:10:30
WENGANM	ILL	10	2.779	94.32	2.92	2.66	737 / 151	83.61	1.692	0.433	18,121	1,630	6,364	152,187	94.54	120	37	11:18:20
WENGANM	ILL	15	2.785	94.44	7.60	5.72	762 / 129	70.43	1.452	0.382	17,836	1,902	7,232	154,877	94.86	137	37	12:21:11
WENGANM	ILL	20	2.787	94.49	11.14	7.39	750 / 118	63.65	1.303	0.361	17,706	2,122	7,655	156,174	94.91	152	37	13:24:43
WENGANM	ILL	25	2.788	94.53	11.68	8.23	758 / 130	59.88	1.219	0.351	17,617	2,271	7,891	157,065	94.91	166	37	14:18:14
WENGANM	ILL	30	2.790	94.56	11.99	8.44	810 / 133	57.93	1.172	0.345	17,779	2,361	8,024	155,616	94.66	170	42	14:23:24
FLYE	ONT	10	2.834	94.29	0.65	0.63	1,768 / 477	938.11	22.375	1.547	17,367	122	1,764	157,179	56.70	281	270	10:15:20
FLYE	ONT	15	2.822	94.90	3.83	3.50	1,644 / 177	560.36	14.035	1.077	17,680	197	2,568	156,518	70.88	370	334	14:01:46
FLYE	ONT	20	2.820	94.96	13.35	10.16	1,887 / 139	425.2	10.610	0.976	17,856	261	2,843	155,358	76.63	482	398	16:15:15
FLYE	ONT	25	2.828	95.07	16.19	13.06	2,459 / 136	363.11	8.943	0.942	17,675	310	2,945	156,984	80.21	615	467	21:04:14
FLYE	ONT	30	2.830	95.10	16.89	13.22	2,591 / 128	328.29	7.995	0.929	17,852	347	2,990	155,540	82.19	738	531	22:00:03

Supplementary Table 13: Fosmid evaluation using a total of 103 Fosmids (3.92Mb) of NA12878. "Closed" refers to the number of Fosmids for which 99.5% of their length aligns to a single locus. The identity and phred-Quality Value (QV) metrics are computed from closed Fosmids only. The common closed Fosmids are the Fosmids closed by all the evaluated genome assemblies (86 Fosmids).

Assembler	Tech	LRC	Closed Fosmid		Fosmid bases		Closed Fosmid				Common closed Fosmid (86)			
							Median Quality		Mean Quality		Median Quality		Mean Quality	
			#	%	length	%	%Identity	QV	%Identity	QV	%Identity	QV	%Identity	QV
WENGANA	MGI	10	89	86.41	3,410,705	86.83	99.77	26.34	99.58	23.76	99.77	26.47	99.59	23.92
WENGANA	MGI	15	94	91.26	3,600,050	91.65	99.82	27.44	99.39	22.15	99.83	27.63	99.65	24.56
WENGANA	MGI	20	94	91.26	3,599,919	91.65	99.83	27.64	99.68	24.89	99.84	27.92	99.67	24.83
WENGANA	MGI	25	95	92.23	3,636,440	92.58	99.83	27.76	99.71	25.35	99.83	27.82	99.72	25.46
WENGANA	MGI	30	95	92.23	3,636,440	92.58	99.83	27.80	99.72	25.46	99.84	28.02	99.72	25.53
WENGANA	ILL	10	94	91.26	3,590,963	91.42	99.86	28.50	99.69	25.10	99.87	28.93	99.74	25.86
WENGANA	ILL	15	95	92.23	3,634,145	92.52	99.87	28.88	99.75	26.02	99.88	29.25	99.76	26.20
WENGANA	ILL	20	96	93.20	3,670,535	93.44	99.88	29.10	99.76	26.28	99.89	29.46	99.77	26.36
WENGANA	ILL	25	96	93.20	3,670,535	93.44	99.87	28.92	99.78	26.49	99.88	29.37	99.78	26.56
WENGANA	ILL	30	96	93.20	3,670,535	93.44	99.88	29.10	99.76	26.27	99.89	29.75	99.77	26.31
WENGAND	MGI	10	96	93.20	3,670,535	93.44	99.89	29.70	99.77	26.29	99.90	29.91	99.78	26.58
WENGAND	MGI	15	96	93.20	3,670,535	93.44	99.90	29.88	99.77	26.44	99.90	29.92	99.78	26.54
WENGAND	MGI	20	96	93.20	3,670,535	93.44	99.90	29.92	99.79	26.70	99.90	30.14	99.79	26.74
WENGAND	MGI	25	96	93.20	3,670,535	93.44	99.90	29.90	99.79	26.76	99.90	30.04	99.79	26.76
WENGAND	MGI	30	96	93.20	3,670,535	93.44	99.90	30.07	99.79	26.82	99.91	30.25	99.79	26.84
WENGAND	ILL	10	96	93.20	3,682,021	93.74	99.89	29.63	99.72	25.60	99.90	30.17	99.77	26.31
WENGAND	ILL	15	97	94.17	3,712,392	94.51	99.90	30.20	99.75	26.03	99.91	30.69	99.77	26.44
WENGAND	ILL	20	97	94.17	3,712,392	94.51	99.91	30.62	99.76	26.24	99.92	31.03	99.78	26.49
WENGAND	ILL	25	97	94.17	3,712,392	94.51	99.90	30.16	99.78	26.51	99.91	30.63	99.79	26.69
WENGAND	ILL	30	96	93.20	3,670,535	93.44	99.91	30.63	99.79	26.87	99.92	30.76	99.80	26.91
WENGANM	MGI	10	93	90.29	3,557,496	90.57	99.84	27.90	99.62	24.15	99.86	28.42	99.65	24.56
WENGANM	MGI	15	96	93.20	3,670,535	93.44	99.85	28.21	99.63	24.37	99.86	28.67	99.65	24.51
WENGANM	MGI	20	96	93.20	3,670,535	93.44	99.86	28.46	99.68	24.94	99.87	29.02	99.68	24.89
WENGANM	MGI	25	96	93.20	3,670,535	93.44	99.88	29.13	99.72	25.52	99.88	29.29	99.72	25.50
WENGANM	MGI	30	96	93.20	3,670,535	93.44	99.88	29.12	99.73	25.65	99.89	29.67	99.72	25.58
WENGANM	ILL	10	93	90.29	3,551,799	90.42	99.87	28.72	99.73	25.71	99.87	29.02	99.74	25.87
WENGANM	ILL	15	94	91.26	3,594,981	91.52	99.88	29.20	99.75	26.00	99.88	29.32	99.75	26.08
WENGANM	ILL	20	95	92.23	3,631,371	92.45	99.87	28.85	99.77	26.29	99.89	29.48	99.77	26.40
WENGANM	ILL	25	95	92.23	3,631,371	92.45	99.88	29.15	99.77	26.43	99.89	29.40	99.78	26.54
WENGANM	ILL	30	95	92.23	3,631,371	92.45	99.88	29.07	99.77	26.43	99.88	29.30	99.78	26.52
FLYE	ONT	10	91	88.35	3,483,942	88.69	98.26	17.60	98.04	17.07	98.27	17.63	98.09	17.18
FLYE	ONT	15	96	93.20	3,676,002	93.58	98.80	19.19	98.71	18.88	98.80	19.20	98.73	18.96
FLYE	ONT	20	98	95.15	3,752,655	95.54	99.05	20.22	98.91	19.62	99.06	20.28	98.94	19.74
FLYE	ONT	25	96	93.20	3,670,535	93.44	99.14	20.67	99.03	20.12	99.16	20.78	99.05	20.20
FLYE	ONT	30	98	95.15	3,752,655	95.54	99.20	20.94	99.09	20.39	99.22	21.08	99.11	20.53

Supplementary Table 14: Polishing the FLYE assembly of NA12878 with short (NovaSeq) and long (ONT flipflop) reads. The FLYE assembly was polished using two rounds of long-read polishing with RACON followed by three rounds of short-read polishing with NTEDIT. The short-read polishing was done using the same short-reads used in the WENGAN assemblies (NovaSeq 53X of pair-end 2x150bp reads). Consensus quality statistics after each round of polishing are presented.

			FLYE	FLYE+ RACON X 1	FLYE+ RACON X 2	FLYE+ RACON X 2+ NTEDIT X 3
T. length (Mb)			2,814.05	2,806.45	2,806.03	2,817.79
Aln. length (Mb)			2,772.60	2,768.34	2,767.77	2,768.09
bases < 99% (Mb)			41.44	38.11	38.26	49.70
Indels	short [1-2]	Number	7,969,035	11,870,633	12,091,389	1,908,590
		Rate (bp)	348	233	229	1,450
	medium [3,50)	Number	925,062	777,251	776,941	429,712
		Rate (bp)	2,997	3,562	3,562	6,442
	large >50	Number	17,395	17,127	17,132	17,180
		Rate (bp)	159,391	161,636	161,556	161,123
Fosmid median QV			21.08	21.68	21.62	27.21
Busco		#Genes	3,373	-	-	3,840
		%Complete	82.19	-	-	93.56
Computational Resources		CPU (h)	-	67	134	368
		RAM (Gb)	-	270	270	270

Supplementary Table 15: WENGAN assemblies of non-human genomes. L50 and L90 metrics are the minimum number of contigs needed to cover 50% and 90% of the genome assembly, respectively. The BUSCO analysis was performed using as database *embryophyta* (1,440 groups), *diptera* (2,799 groups), and *actinopterygii* (4,584 groups) for assessing WENGAN assemblies of *Arabidopsis thaliana*, *Drosophila mojavensis*, and *Thamnaconus septentrionalis*, respectively. The BUSCO complete genes are reported. All WENGAN assemblies were run using 20 CPUs.

	Total length (bp)	Number	Longest (bp)	N50 (bp)	L50	N90 (bp)	L90	BUSCO complete (%)	CPU (h)	RAM (Gb)	Elapsed h:m:s
Arabidopsis thaliana (Plant)											
WENGANA (ONT)	117,506,078	132	12,970,918	9,090,558	6	1,175,083	18	98.2	30	24	1:37:16
WENGANA (PAC)	118,529,634	62	16,182,978	12,781,507	5	1,722,142	10	98.1	34	24	1:49:35
WENGAND (ONT)	118,716,946	127	14,075,903	9,426,751	5	1,182,659	18	98.2	16	125	1:08:40
WENGAND (PAC)	119,426,674	82	16,200,101	12,773,488	5	1,704,574	13	98.3	19	125	1:19:09
WENGANM (ONT)	116,741,168	158	14,409,767	9,394,408	5	677,048	20	98.3	6	7	0:32:05
WENGANM (PAC)	117,786,141	95	14,435,119	9,400,311	5	1,443,075	18	98.3	8	7	0:38:08
Drosophila mojavensis (Insect)											
WENGANA (ONT)	151,700,204	264	28,214,474	11,881,786	4	302,618	49	97.4	35	24	2:00:00
WENGAND (ONT)	154,707,097	137	26,452,207	25,667,050	3	2,495,673	8	98.3	33	139	2:04:18
WENGANM (ONT)	154,260,731	214	20,472,051	11,882,057	5	1,519,782	18	98.3	18	7	1:20:26
Thamnaconus septentrionalis (Fish)											
WENGANA (ONT)	471,216,505	204	31,551,066	15,775,403	12	1,976,564	41	95.0	96	45	5:50:10
WENGAND (ONT)	476,028,467	218	21,865,951	14,362,854	13	2,824,309	36	95.8	288	148	17:13:59
WENGANM (ONT)	476,651,601	487	21,993,416	14,251,541	13	1,952,617	44	94.8	57	32	7:07:14

Supplementary Table 16: BAC and Fosmid sequences used to assess the consensus accuracy of genome assemblies. The sequences of NA12878 were obtained by randomly selecting 103 clones from a NA12878 Fosmid library. The BAC sequences of HG0073 and CHM13 were obtained from a BAC library enriched in segmental duplications.

Sample	Sequences	Type	Total	Length (Mb)
NA128978	NA12878_clones.ver_1.0.fasta	Fosmid	103	3.92
HG0073	HG0073-BACs.fasta	BAC	179	27.96
CHM13	CHM13-BACs.fasta	BAC	341	51.53

Supplementary Table 17: Short and long reads datasets of non-human genomes. Public genomic data from a plant (*Arabidopsis thaliana*), an insect (*Drosophila mojavensis*), and a fish (*Thamnaconus septentrionalis*) were collected and assembled using the three WENGAN modes.

	Technology	Read count	Coverage	Pairs / N50	Platform	SRA / ENA
Arabidopsis thaliana	Illumina	33,683,902	70X	2x250 bp	MiSeq	ERR2173372
	ONT	297,234	30X	20,132	MinION	ERR2173373
	PacBio	573,444	60X	20,031	Sequel I	ERR2173371
Drosophila mojavensis	Illumina	82,467,984	80X	2x151 bp	NextSeq 500	SRR6425997
	ONT	1,315,872	50X	10,605	MinION	SRR7167955
Thamnaconus septentrionalis	Illumina	306,820,704	95X	2x150 bp	HiSeq X Ten	SRR10134766
	ONT	19,342,211	176X	10,567	PromethION	SRR10150407

Genomic Location	Query name	Query start	Query end	Length	Score	E-val	%ID
8:121868733-121879155 [Sequence]	10072	1	10423	10423 [Sequence]	20092.0	0.0e+00	99.87 [Alignment]
7:145426428-145434392 [Sequence]	10072	10422	18386	7965 [Sequence]	15432.0	0.0e+00	99.94 [Alignment]

HSP distribution on genome

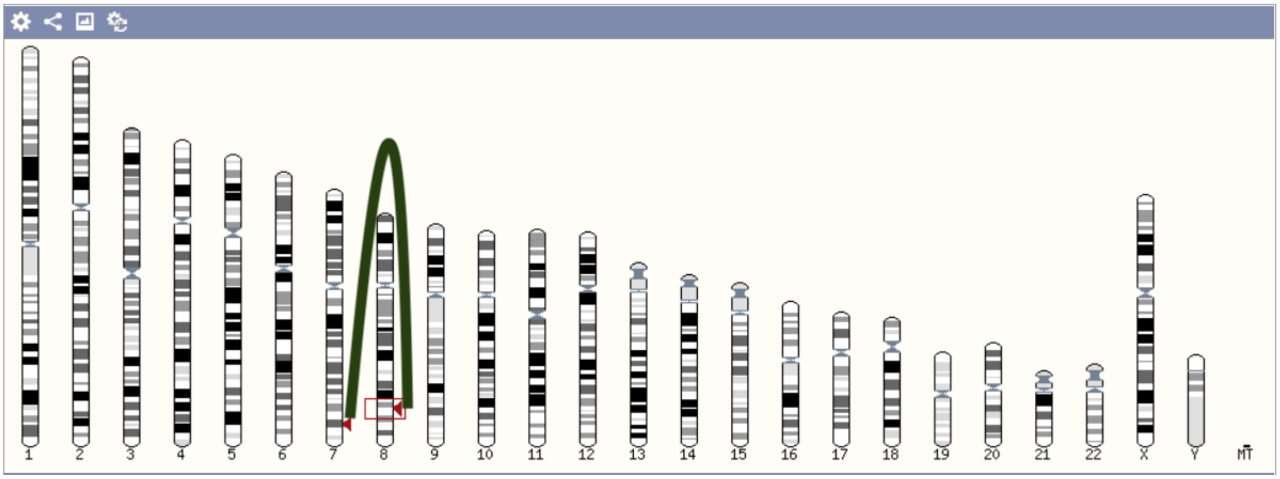

Supplementary Figure 1: Example of a chimeric contig detected by INTERVALMISS on the MINIA3 assembly of NA12878. INTERVALMISS identifies a lack of fragment coverage starting at base position 10,434 and ending at base position 10,540 of the contig 10072. A BLAST search on the human reference genome confirms the breakpoint occurring at the contig interval 10,422-10,423. In this case, the chimeric contig induces an erroneous inter-chromosome translocation between chromosomes 8 and 7. INTERVALMISS splits the chimeric MINIA3 contig at the flanking positions of the interval [10,433-10,540], originating in two new subcontigs covering the positions 1-10,432 and 10,541-18,386, and solving the breakpoint. The figure was generated using the ENSEMBL BLAST portal [https://www.ensembl.org/Homo_sapiens/Tools/Blast].

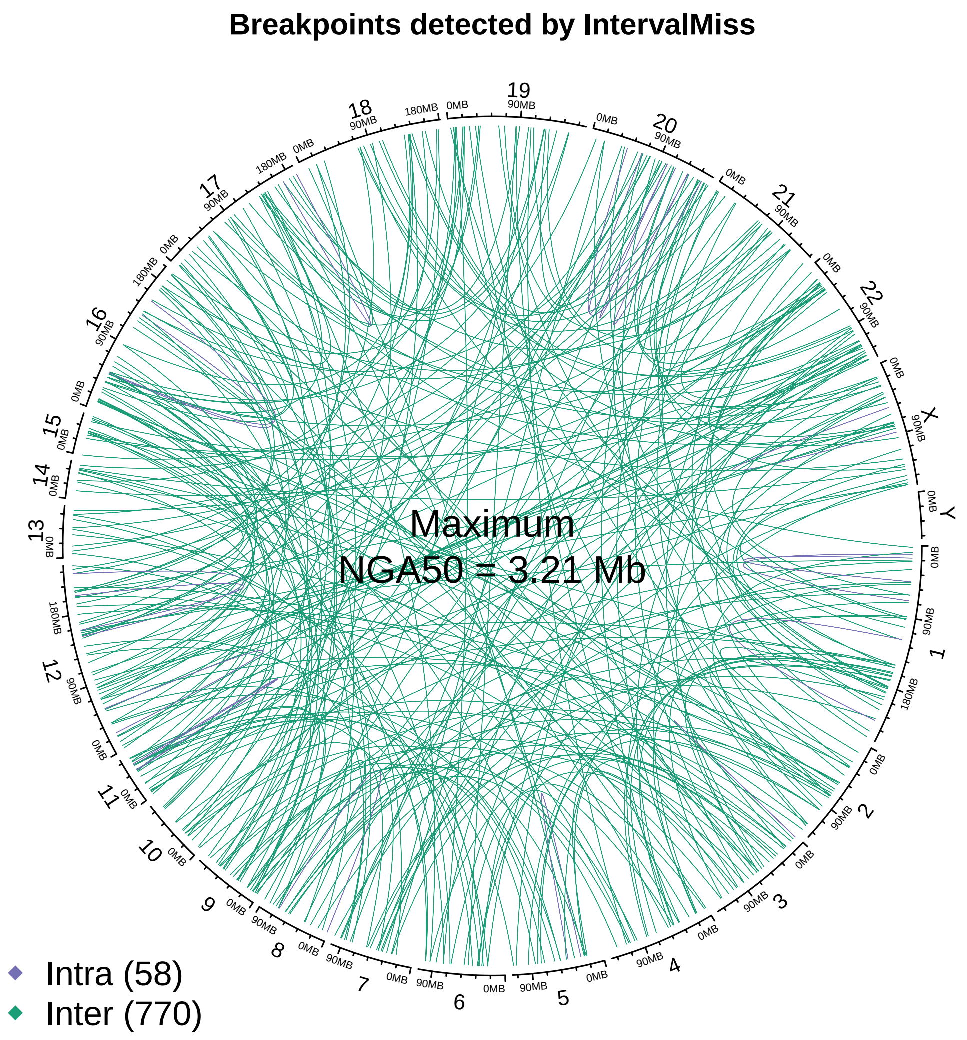

Supplementary Figure 2: The circular plot depicts the number of missassemblies detected using the pair-end information and the maximum NGA50 that can be achieved if those contigs are not corrected in the NA12878 MINIA3 short-read assembly.

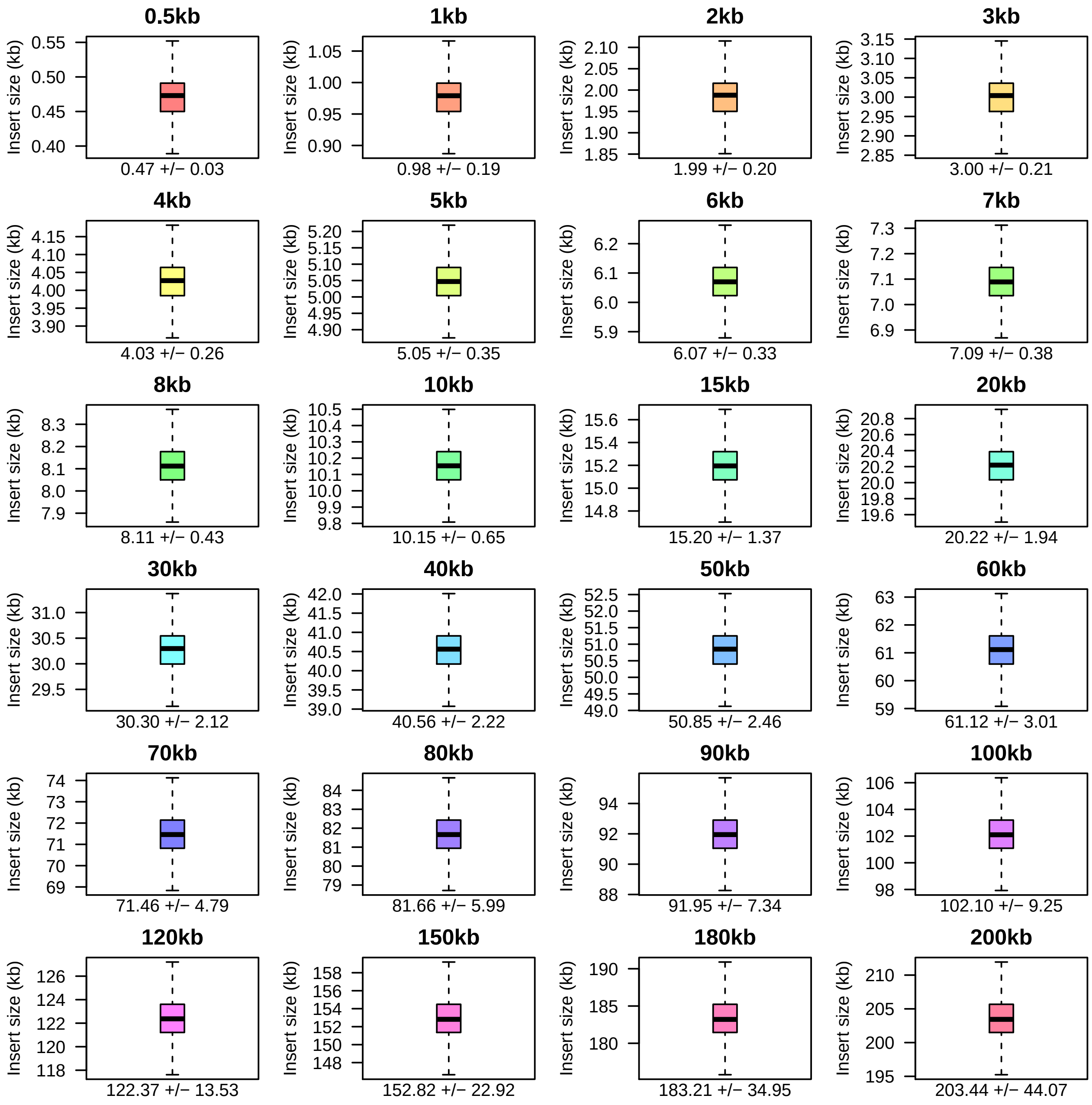

Supplementary Figure 3: Spectrum of synthetic mate-pair libraries generated by FastMin-SG from ultralong Nanopore reads of NA12878 (rel5). A maximum of 900,000 insert sizes were collected from the mate-pair reads aligned by FastMin-SG within contigs of the NA12878 (Discovar Assembly). A total of n=900,000 insert sizes were collected for libraries in the range of 0.5 to 120kb. A total of n=549,784, n=269,453, and n=169,884 insert sizes were collected for the 150kb, 180kb and 200kb libraries, respectively. The percentage of outlier synthetic pairs detected ranged from a minimum of 1.18% (0.5kb) to a maximum of 16.17% (200kb). The boxplots were drawn excluding outlier synthetic pairs. The median and the standard deviation are depicted below each boxplot (median +/- sd).

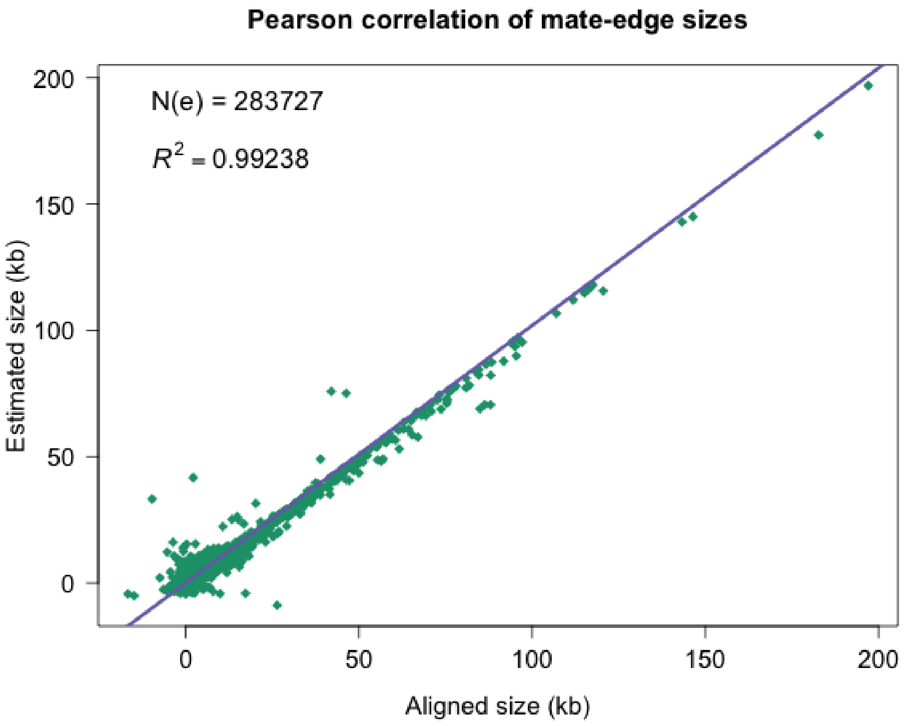

Supplementary Figure 4: The Pearson correlation of the mate-edge lengths before (y-axis) and after (x-axis) building the consensus sequences for a total of n=283,727 mate-edges from the NA12878 WENGAN$_M$ assembly is depicted. Notice that the agreement between the estimated and the aligned mate-edge lengths exceeds $R^2 > 0.99$.

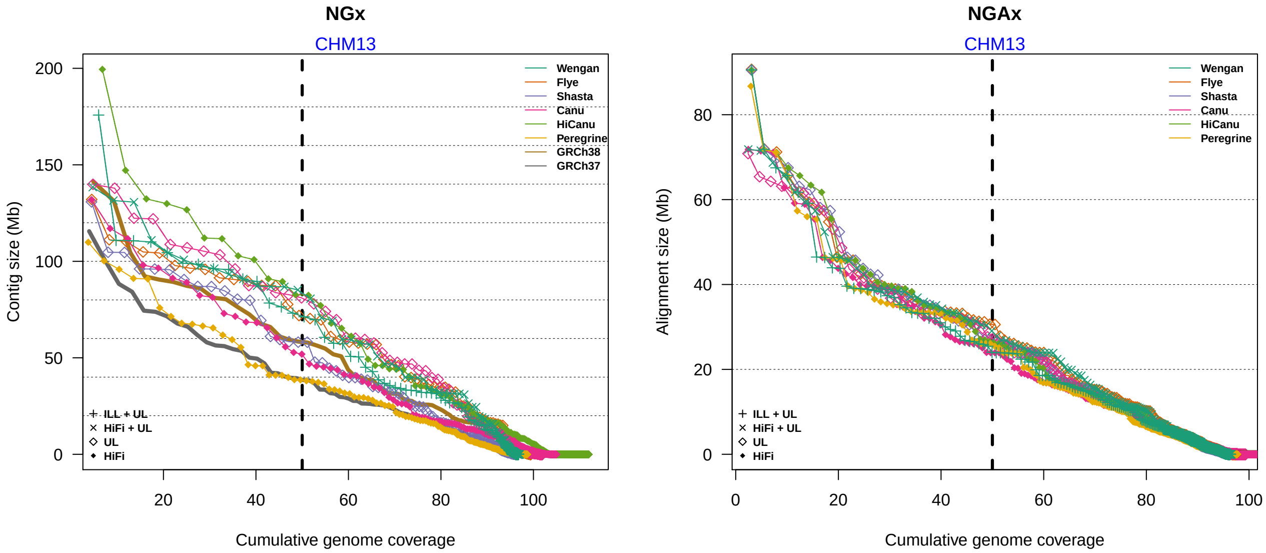

Supplementary Figure 5: QUAST NGx and NGAx of CHM13 assemblies.

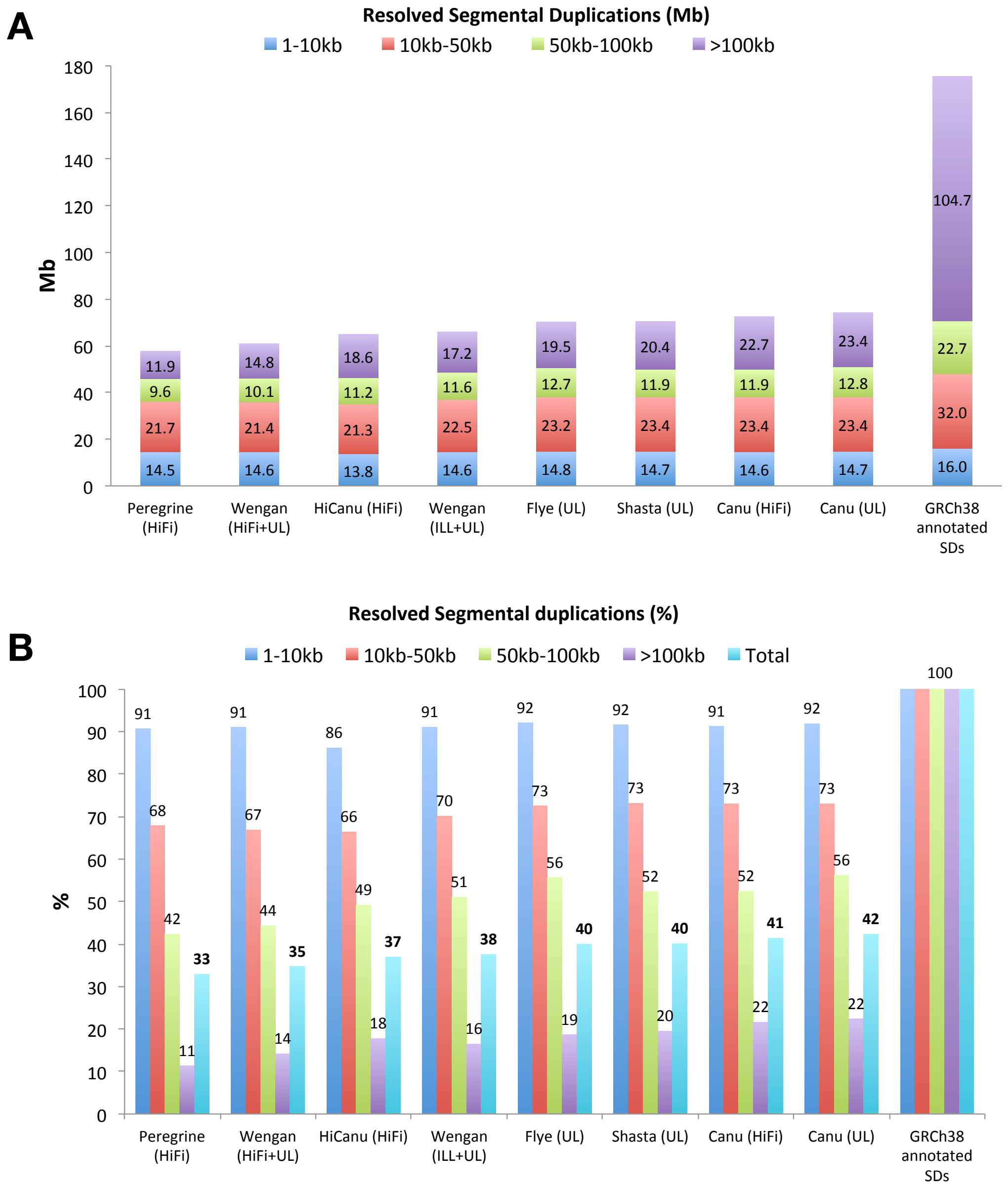

Supplementary Figure 6: Segmental Duplications (SD) resolved by different genome assemblies of CHM13. An SD is considered resolved if the aligned contig extends the SD flanking sequences by at least 50kb. A total of n=8,048 SDs with a total sequence length of 175.4Mb were assessed. A) The stacked plot displays the amount (Mb) of SD sequences resolved binned by length (1-10kb,10-50kb,50-100kb,>100kb) for the assemblies and the GRCh38 reference genome. B) The barplot displays the percentage of SDs resolved binned by length relative to the GRCh38 reference.

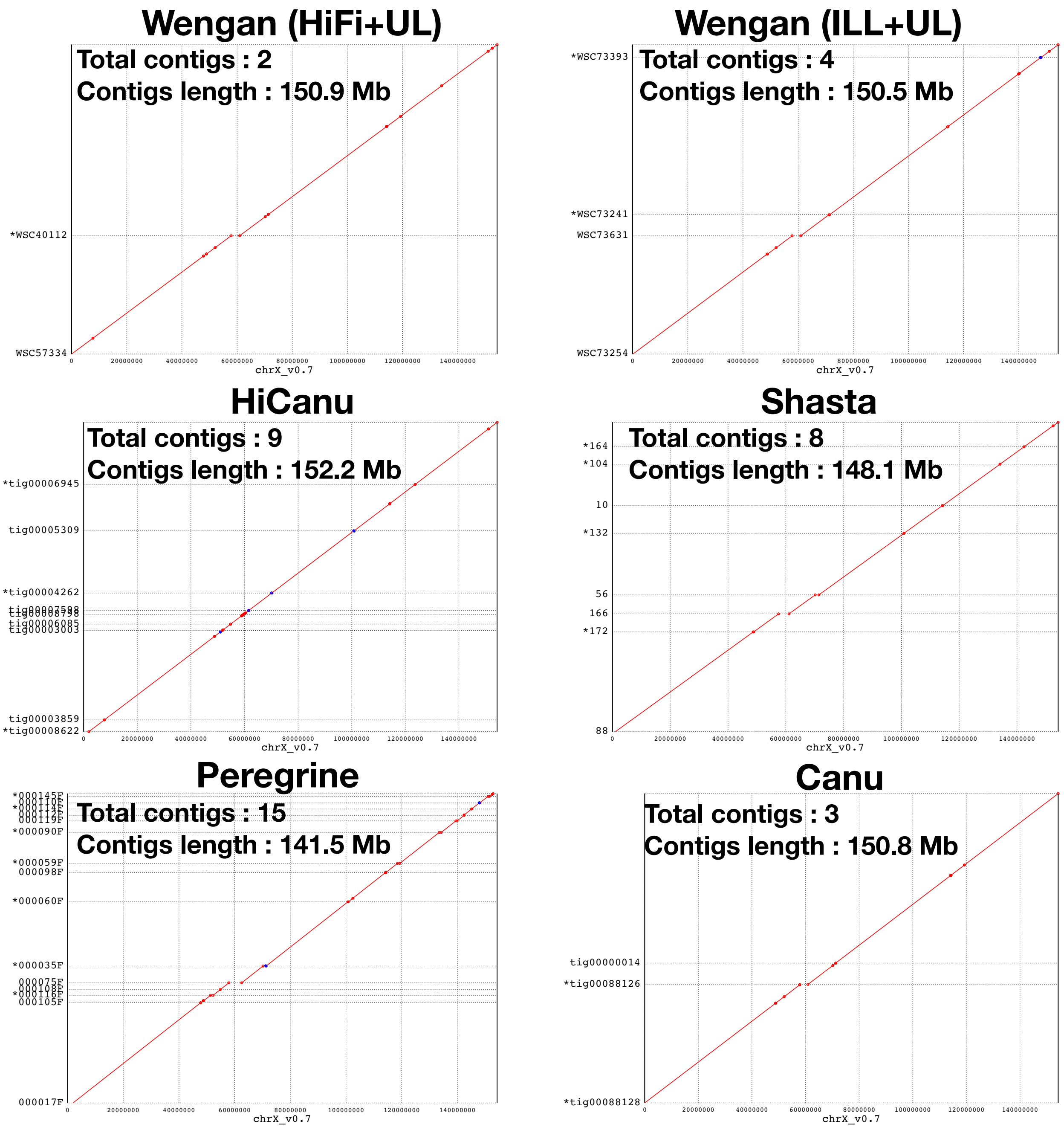

Supplementary Figure 7: Assembled contigs were aligned to the T2T-X chromosome using MASH version 2.0 (" -r chrX.t2t.fa -f one-to-one -q asm.fa -s 10000 –pi 85"). Contigs with an alignment block >= 1Mb at an average identity >= 98% were anchored to the CHM13 T2T-X chromosome (v.07). Anchored contigs were then masked using REPEATMASKER.

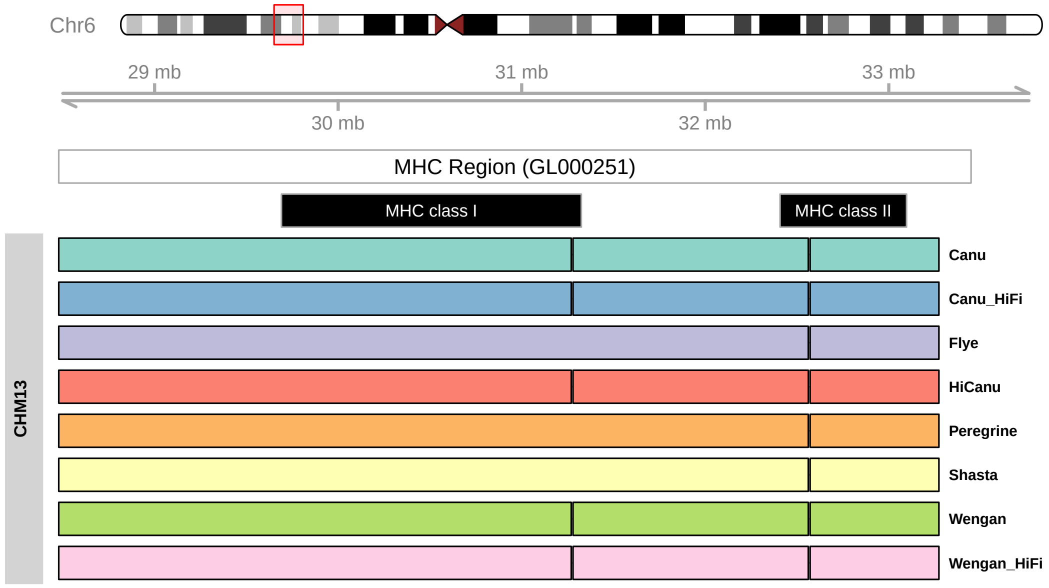

Supplementary Figure 8: All the evaluated assemblers span the MHC region in a single contig. The SHASTA, PEREGRINE and FLYE assemblies reach the higher NGA50 with a value of 4.08Mb. The WENGAN and CANU assemblies reach an NGA50 of 2.79Mb. The WENGAN(HIFI+UL) contig that spans the MHC region is WSC27686[1,636,665-6,427,628], with a total length of 31.5Mb. The sequence of the GL000251.2 haplogroup was used as the closed reference for CHM13. The GL000251.2 sequence was aligned to the genome assemblies and the aligned blocks $\geq$ 30kb with a minimum identity of 95% were kept. The alignment breakpoints (vertical black lines) indicate a contig switch, alignment error or gap in the assembly.

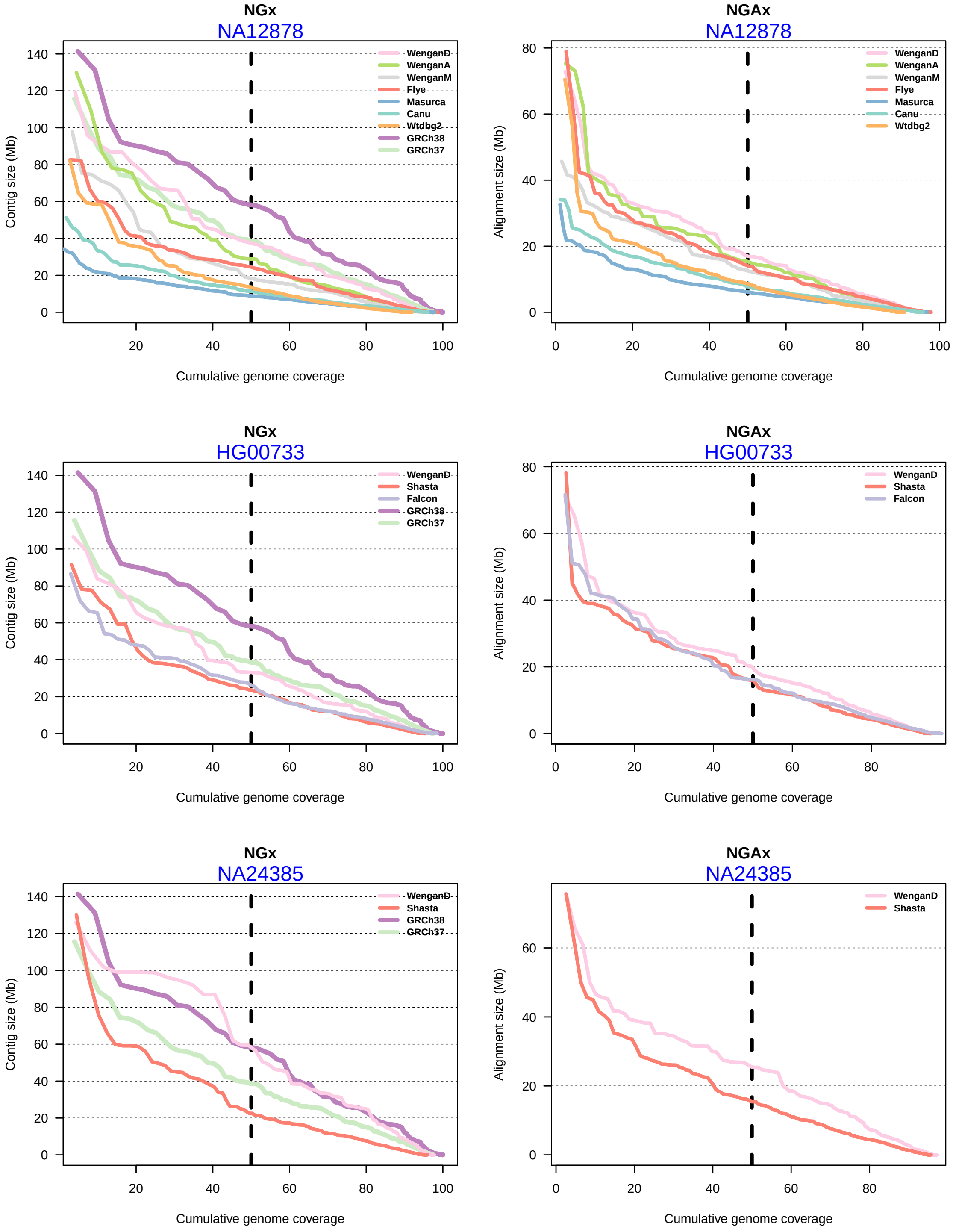

Supplementary Figure 9: QUAST NGx and NGAx of NA12878, HG00733, and NA24385 assemblies.

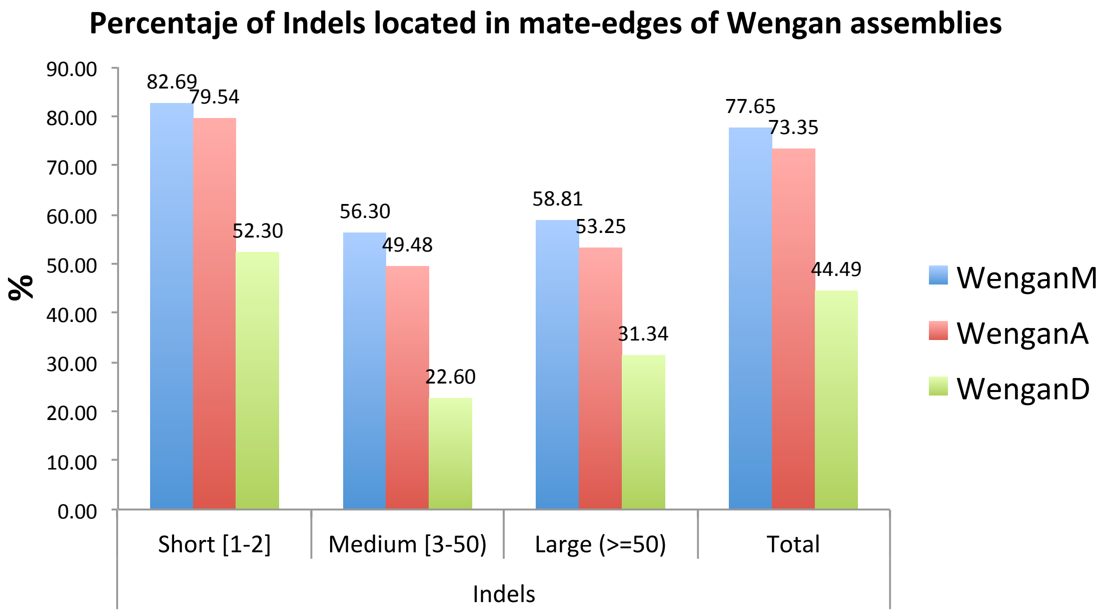

Supplementary Figure 10: Distribution of the WENGAN consensus errors of the hybrid assemblies of NA12878 generated with Illumina (2x150 and 2x250) and ultralong Nanopore reads (rel5). The total number of short, medium and large indels are 1,990,947; 454,484; 17,954 for WENGANM, 1,722,477; 431,273; 18,733 for WENGANA, and 854,330; 294,128; 17,744 for WENGAND. The majority of the consensus errors are located in the long-read consensus sequences of mate-edges. The size of such sequences ranges from 80Mb (WENGAND) to 270Mb (WENGANM) per assembly, thus reducing the amount of sequence to be polished by at least 90%.

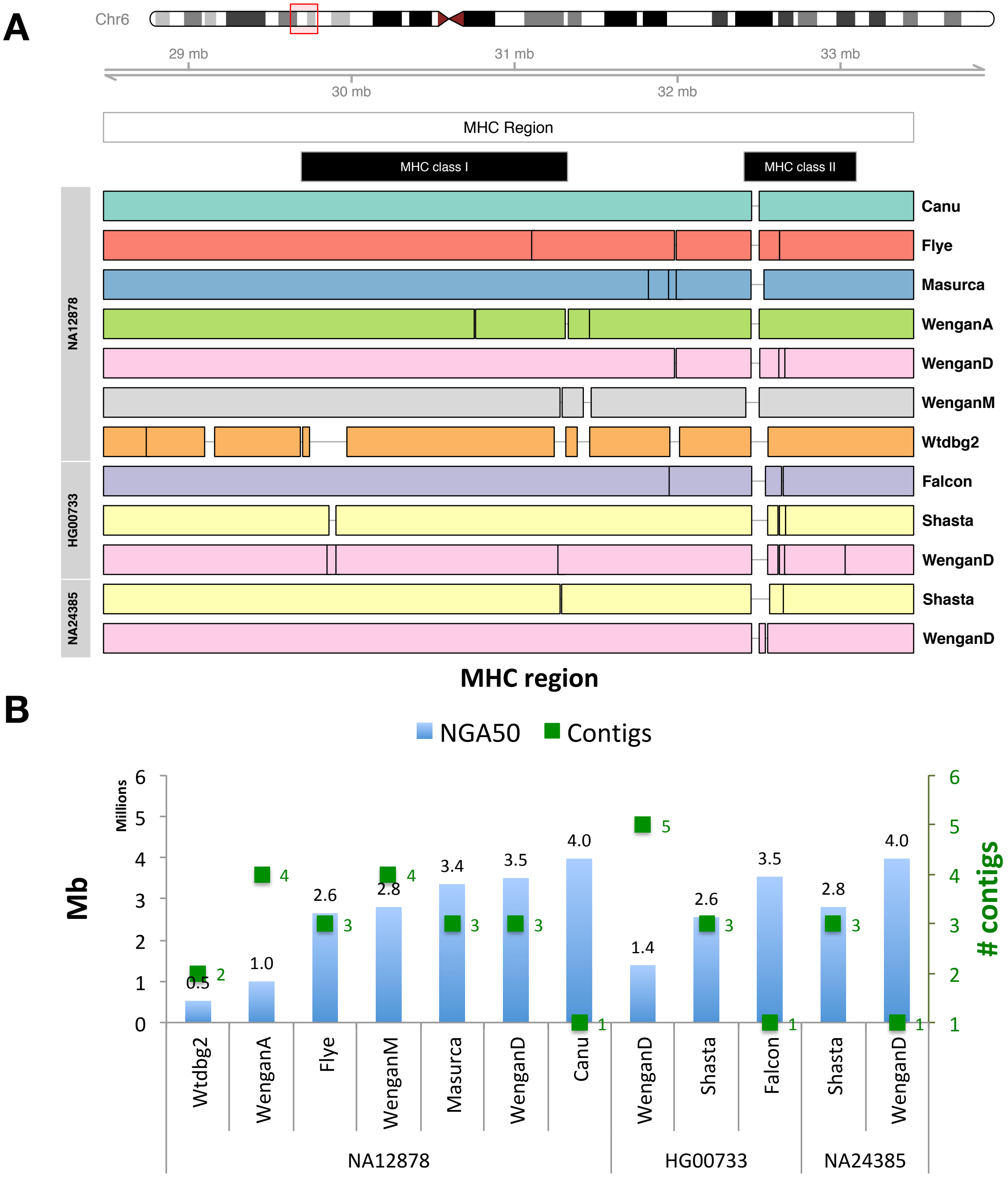

Supplementary Figure 11: Assembly of the complex MHC region in NA12878, HG00733 and NA24385 genomes. A) The MHC sequence was aligned to the genome assemblies and the aligned blocks $\geq$ 30kb with a minimum identity of 95% were kept. The alignment breakpoints (vertical black lines) indicate a contig switch, alignment error or gap in the assembly. B) The NGA50 and the number of contigs spanning the MHC region of each diploid assembly are depicted. NGA50 is NG50 corrected of assembly errors. The NGA50 was computed using a genome size equal to the length of the MHC region (n=4.97Mb).

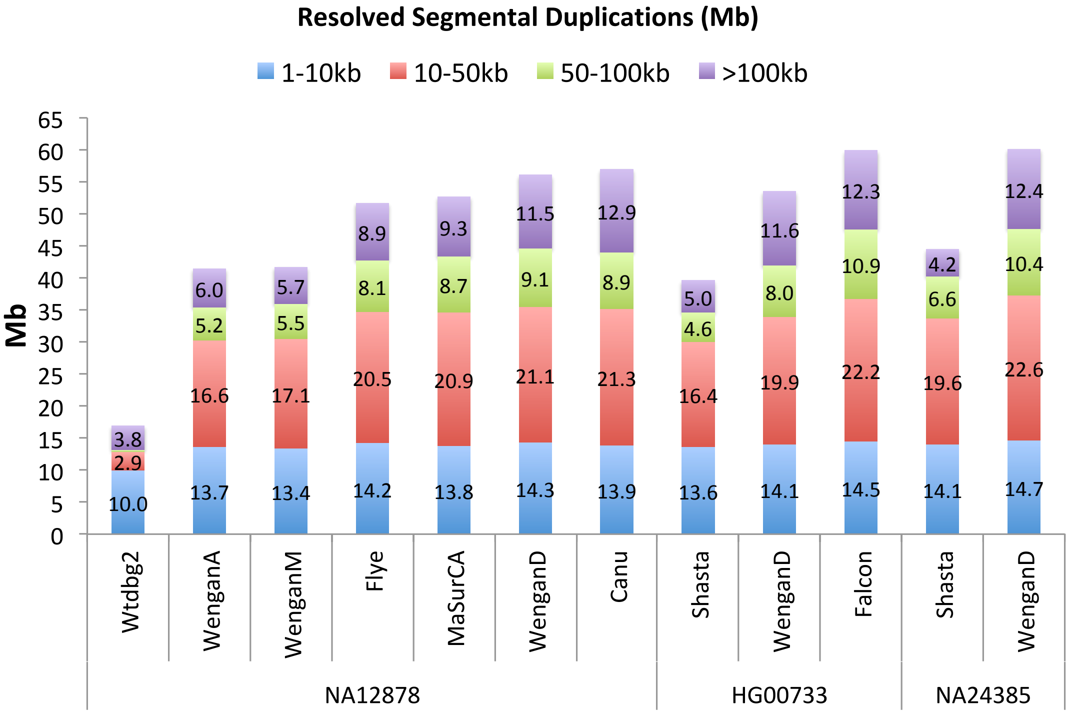

Supplementary Figure 12: An SD is considered resolved if the aligned contig extends the SD flanking sequences by at least 50kb. A total of n=8,048 SDs with a total sequence length of 175.4Mb were assessed. The stacked plot displays the amount (Mb) of SD sequences resolved binned by length (1-10kb,10-50kb,50-100kb,>100kb) for each assembly.

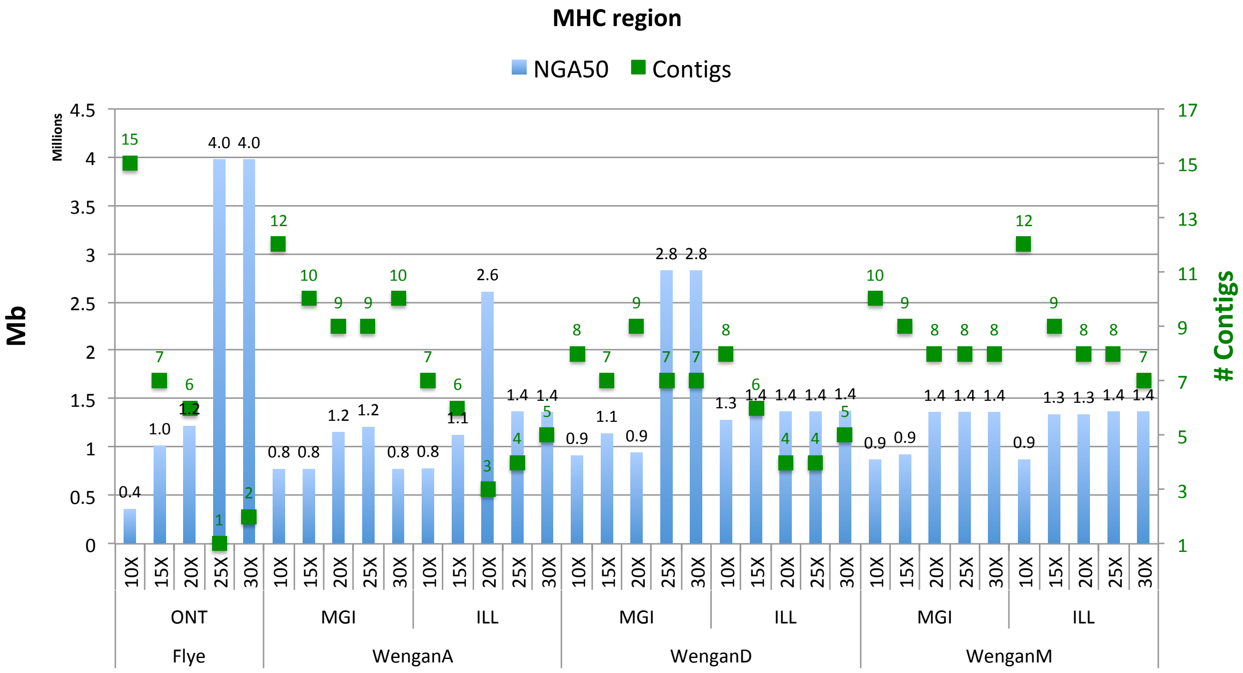

Supplementary Figure 13: Assembly of the complex MHC region at different long-read coverage. The NGA50 and the number of contigs spanning the MHC region of each assembly of NA12878 are depicted. NGA50 is NG50 corrected of assembly errors. The NGA50 was computed using a genome size equal to the length of the MHC region (n=4.97Mb).
