# Supplementary material for: Efficient hybrid de novo assembly of human genomes with WENGAN
Source: Nat Biotechnol. 2020 Dec 14;39(4):422–30. doi: 10.1038/s41587-020-00747-w (PMC8041623; doi:10.1038/s41587-020-00747-w)
Supplement: Supplementary file 2 — Reporting Summary [file 41587_2020_747_MOESM2_ESM.pdf]

nature research

Corresponding author(s): Alex Di Genova

Last updated by author(s): Sep 28, 2020

Reporting Summary

Nature Research wishes to improve the reproducibility of the work that we publish. This form provides structure for consistency and transparency in reporting. For further information on Nature Research policies, see Authors & Referees and the Editorial Policy Checklist.

Statistics

For all statistical analyses, confirm that the following items are present in the figure legend, table legend, main text, or Methods section.

n/a	Confirmed	
☒	☐	The exact sample size (*n*) for each experimental group/condition, given as a discrete number and unit of measurement
☒	☐	A statement on whether measurements were taken from distinct samples or whether the same sample was measured repeatedly
☒	☐	The statistical test(s) used AND whether they are one- or two-sided<br>*Only common tests should be described solely by name; describe more complex techniques in the Methods section.*
☒	☐	A description of all covariates tested
☒	☐	A description of any assumptions or corrections, such as tests of normality and adjustment for multiple comparisons
☒	☐	A full description of the statistical parameters including central tendency (e.g. means) or other basic estimates (e.g. regression coefficient) AND variation (e.g. standard deviation) or associated estimates of uncertainty (e.g. confidence intervals)
☒	☐	For null hypothesis testing, the test statistic (e.g. *F*, *t*, *r*) with confidence intervals, effect sizes, degrees of freedom and *P* value noted<br>*Give P values as exact values whenever suitable.*
☒	☐	For Bayesian analysis, information on the choice of priors and Markov chain Monte Carlo settings
☒	☐	For hierarchical and complex designs, identification of the appropriate level for tests and full reporting of outcomes
☒	☐	Estimates of effect sizes (e.g. Cohen's *d*, Pearson's *r*), indicating how they were calculated

Our web collection on statistics for biologists contains articles on many of the points above.

Software and code

Policy information about availability of computer code

Data collection	No custom software were used for data collection.
Data analysis	The manuscript presents a de novo genome assembler called Wengan. The code is freely available at https://github.com/adigenova/wengan. Additional data, such as genome assemblies that were generated in our analysis are available at https://zenodo.org/record/3779515.. List of software used in the manuscript: Wengan v0.2, Minia3 (commit 017d23e), Abyss2 v2.1.5, DiscovarDenovo version discovarexp-51885, Flye v2.5, Minimap2 v2.15-r905, Paftools v2.15-r905, Racon v1.4.9, NTedit v1.2.3, Kmc v3.1.0, Quast v5.0.2, Busco v3.0.2, bacValidation (commit b179af4), SegDupPlots (commit d1394cf), RepeatMasker (4.1.0), Mashmap (version v2.0), and Bionano Solve v3.4_06042019a.

For manuscripts utilizing custom algorithms or software that are central to the research but not yet described in published literature, software must be made available to editors/reviewers. We strongly encourage code deposition in a community repository (e.g. GitHub). See the Nature Research guidelines for submitting code & software for further information.

Data

Policy information about availability of data

All manuscripts must include a data availability statement. This statement should provide the following information, where applicable:

- Accession codes, unique identifiers, or web links for publicly available datasets
- A list of figures that have associated raw data
- A description of any restrictions on data availability

All sequence datasets and de novo genome assemblies described in the manuscript are publicly available through the corresponding repositories. Specific hyperlinks for the four human datasets are provided in the Supplementary Material: Supplementary Table 1 provides hyperlinks for all the long-read datasets; Supplementary Table 2 provides hyperlinks for all the short-read datasets; Supplementary Table 3 provides hyperlinks for all the de novo assemblies used in the benchmark; Supplementary Table 16 provides hyperlinks for the BAC/Fosmid sequences used for consensus quality assessment. The BIONANO data of CHM13 is available at https://github.com/nanopore-wgs-consortium/CHM13. Specific hyperlinks for the non-human datasets are provided in the Supplementary Table 17. The

October 2018

supplementary files, including all the WENGAN assemblies described in the present manuscript, are available through Zenodo at https://zenodo.org/record/3779515. The specific commands for each WENGAN assembly are provided in the Supplementary Material (Subsection 1.2). The NovaSeq6000, MGISEQ-2000RS and PromethION sequence data of NA12878 were submitted to the Sequence Read Archive (SRA) under the BioProject PRJNA603060.

Field-specific reporting

Please select the one below that is the best fit for your research. If you are not sure, read the appropriate sections before making your selection.

☒ Life sciences ☐ Behavioural & social sciences ☐ Ecological, evolutionary & environmental sciences

For a reference copy of the document with all sections, see nature.com/documents/nr-reporting-summary-flat.pdf

Life sciences study design

All studies must disclose on these points even when the disclosure is negative.

Sample size	Not applicable, since a single sample was sequenced.
Data exclusions	No data was excluded from the analysis.
Replication	Not applicable, since the manuscript describes deterministic algorithms. We have references all publicly available datasets and software versions for reproducibility of our analysis.
Randomization	Not applicable.
Blinding	Not applicable, since our manuscript does not require case/control comparison.

Reporting for specific materials, systems and methods

We require information from authors about some types of materials, experimental systems and methods used in many studies. Here, indicate whether each material, system or method listed is relevant to your study. If you are not sure if a list item applies to your research, read the appropriate section before selecting a response.

Materials & experimental systems

n/a	Involved in the study	
☒	☐	Antibodies
☐	☒	Eukaryotic cell lines
☒	☐	Palaeontology
☒	☐	Animals and other organisms
☒	☐	Human research participants
☒	☐	Clinical data

Methods

n/a	Involved in the study	
☒	☐	ChIP-seq
☒	☐	Flow cytometry
☒	☐	MRI-based neuroimaging

Eukaryotic cell lines

Policy information about cell lines

Cell line source(s)	This study uses NA12878 cell line and DNA which is supplied by Coriell and is approved for genome sequencing governed by the Coriell Institutional Review Board ("Coriell IRB") in accordance with DHHS regulations (45 CFR Part 46) and is not considered human subjects research.
Authentication	Purchased directly from validated source and sequenced. Coriell validates cells as described here: https://www.coriell.org/0/pdf/CC_Process_Flow.pdf
Mycoplasma contamination	Coriell routinely screen the cells for mycoplasma: https://www.coriell.org/0/pdf/CC_Process_Flow.pdf
Commonly misidentified lines (See ICLAC register)	No commonly misidentified cell lines were used.
